# Spray drying: From a traditional technology to modern biotechnological applications

**DOI:** 10.1016/j.ijpx.2025.100449

**Published:** 2025-11-17

**Authors:** Andrea Milanesi, Giada Diana, Alessandro Candiani, Alessandro Sodano, Paolo Rassè, Andrea Foglio Bonda, Elia Bari, Maria Luisa Torre, Lorena Segale, Lorella Giovannelli

**Affiliations:** a Department of Pharmaceutical Sciences, University of Piemonte Orientale, Largo Donegani 2, 28100 Novara, Italy; b APTSol S.r.l., Corso Milano 17, 28100 Novara, Italy

**Keywords:** Spray drying, Powder formulations, Particle engineering, Vaccines, Nanomedicine, Aseptic processing

## Abstract

Spray drying, first patented in the late 19^th^ century, has evolved into a versatile technology for converting liquid feeds into stable, free-flowing powders. Its fundamental strength lies in the rapid atomization and solvent evaporation. It enables precise control over particle size, morphology, and moisture content, making it a consolidated tool across food, chemical, and pharmaceutical industries. In the pharmaceutical field, it has been successfully applied to inhalable powders, amorphous solid dispersions, and controlled-release systems. At the same time, innovations in equipment design and Quality by Design strategies have improved robustness and scalability. Emerging applications now highlight its potential to stabilize biopharmaceuticals and vaccines, where dry powder formulations can enhance shelf life and reduce reliance on the cold chain. Similarly, spray drying has become central in nanomedicine through “nano-into-micro” engineering strategies that transform nanoscale carriers into inhalable or targeted dry powders. A further frontier is aseptic spray drying, which addresses sterility requirements for parenteral formulations and vaccines, representing a key step toward industrial adoption. This review outlines the fundamental principles of spray drying, examines the impact of formulation components, and discusses challenges in scale-up and industrial implementation. It then explores the most recent advances in biopharmaceuticals, nanomedicine, and aseptic processing, offering an integrated perspective on how spray drying is transitioning from a traditional drying method into a platform technology for modern biotechnology and pharmaceutical applications.

## Graphical abstract

**Figure:**
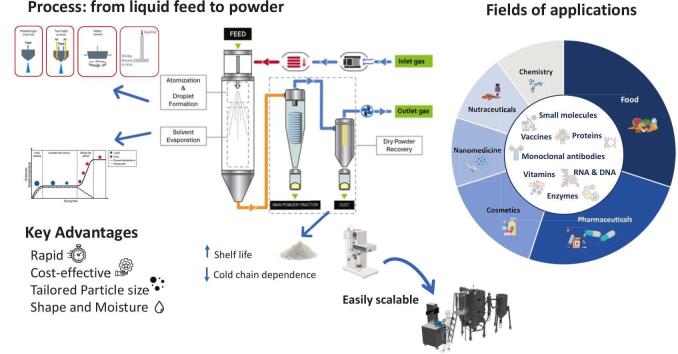

## 1 Introduction

Spray drying, first patented in 1872 by Samuel Percy (Percy, 1872), has become a cornerstone of industrial drying processes, especially in the food, nutraceutical, and pharmaceutical sectors. In the 1920s, driven by the dairy industry, spray drying gained widespread industrial application; the need for large quantities of milk powder during World War II accelerated advancements in this technology, and further improvements continued in the post-war period as milk production surged. In the last decades, spray drying has become a critical technique across different industrial sectors, establishing itself as one of the most effective methods for producing fine, stable, and homogeneous powders (Killeen, 2001; Lechanteur and Evrard, 2020). It is nowadays essential for producing a wide array of products, including food powders, nutraceuticals, probiotics, enzymes, and antibiotics (Jain et al., 2011; Akbarbaglu et al., 2021; Samborska et al., 2022).

This drying technique has thus gradually come to play a vital role in the pharmaceutical industry. Its introduction into pharmaceuticals marked a turning point in the production of inhalable powders, thanks to its ability to precisely control particle size distribution (PSD) and morphology, crucial for ensuring proper drug delivery and bioavailability (Alhajj et al., 2021; Chang and Chan, 2022; Poozesh et al., 2025).

Spray drying has also emerged as a central technique for producing amorphous solid dispersions (ASDs), particularly for addressing the challenges posed by poorly water-soluble drugs (Singh and Van den Mooter, 2016). ASDs stabilize the amorphous state of Active Pharmaceutical Ingredients (APIs) by dispersing them in a polymer matrix, preventing crystallization during storage, and significantly enhancing their solubility and dissolution rates, which is critical for improving bioavailability. Spray drying's ability to achieve this at a large scale while maintaining precise control over particle size, morphology, and moisture content has made it the method of choice for many pharmaceutical applications. As pharmaceutical pipelines increasingly feature complex and low-solubility molecules, the continuous refinement of ASDs via spray drying techniques will be crucial in addressing these challenges, ensuring the development of effective, stable, and scalable drug formulations (Baumann et al., 2021; Wang et al., 2021).

Moreover, spray drying has also demonstrated its effectiveness in obtaining controlled-release drug delivery systems by encapsulating APIs in biodegradable and biocompatible materials (Palmieri et al., 2001; Leena et al., 2020; Nikjoo et al., 2023). For instance, chitosan microparticles produced by spray drying have been successfully used for the controlled release of highly soluble drugs, like venlafaxine hydrochloride, thereby enhancing therapeutic efficacy by extending the release duration (Aranaz et al., 2017).

Spray drying has become an indispensable technique in the pharmaceutical industry because it adheres to recommended guidelines and incorporates the Quality by Design (QbD) approach, ensuring that all critical process parameters are optimized and controlled to achieve consistent product quality. The Pharmaceutical guidelines, particularly the ICH Q8 guidelines, emphasize the importance of the QbD approach, a systematic and scientific method for developing pharmaceutical processes to ensure product quality. This begins by identifying Critical Quality Attributes (CQAs) and Critical Process Parameters (CPPs) that influence the final quality of the product. The goal is to develop processes that consistently meet predefined objectives through sound science and risk management. In the context of spray drying, the QbD approach focuses on optimizing process parameters like inlet/outlet temperatures, feed rate, atomization conditions, and gas flow rates (Maltesen et al., 2008; Nair et al., 2019; Milanesi et al., 2022; Alhajj et al., 2023). These factors are mapped out within a Design Space (DS), which represents the acceptable operational ranges where the product quality remains stable. This ensures that the process is robust, minimizing variability while optimizing performance. The FDA's guidelines also recommend integrating Process Analytical Technology (PAT) tools, such as in-line sensors and real-time monitoring, to dynamically control CPPs during manufacturing, ensuring the final product meets its quality specifications without post-production testing. This approach not only improves efficiency but also reduces the likelihood of product failure during scale-up (Dobry et al., 2009). For these reasons, an in-depth knowledge of the technology, including the equipment and process parameters, is equally essential to understanding the product and formulation.

In this review, the fundamental operation and main components of spray drying are outlined, with a focus on atomization, solvent evaporation, and powder recovery. The influence of the different formulation components – such as sugars, polymers, proteins, lipids, and polysaccharides – on the process and the quality of the final product is examined. Building on this, the challenges of scale-up and industrial manufacturing are discussed, with modern applications in biopharmaceuticals, nanomedicine, and aseptic processing explored, and future perspectives highlighted, concluding with an outlook on the evolving role of spray drying in biotechnology and pharmaceuticals.

## 2 Principles of spray drying

Spray drying is a single-step powder-manufacturing process for converting liquid feeds into dry products: the essence lies in its ability to achieve rapid drying through atomization, where a liquid is transformed into fine droplets and subjected to a stream of hot air, resulting in the formation of dry particles (Refstrup et al., 2016; Ziaee et al., 2019). While the process may seem a straightforward method for producing powders, the kinetics of droplet drying and the subsequent thermal events are quite complex (Zbicinski et al., 2002).

The liquid feed, which can be a solution, emulsion, or suspension, enters the spray dryer and undergoes multiple transformations. Initially, the liquid pushed by a pump reaches the atomizer, which sprays it, creating fine droplets that maximize surface area for efficient drying. The droplets come into contact with a stream of hot air (drawn into the system by a blower and heated at the desired temperature), triggering the drying process within the drying chamber. Each droplet is first heated to the wet-bulb temperature, and the drying begins at the droplet's surface, with solvent molecules migrating outward. During this initial phase, known as the constant-rate drying stage, adiabatic evaporation takes place. As the drying progresses, the solvent content diminishes, leading to the second phase: the falling-rate period. In this stage, a solid crust forms on the surface of the droplet, slowing the drying rate and causing the droplet's temperature to rise until it matches the surrounding gas temperature. The drying air, carrying moisture and dry particles, moves toward the bottom of the chamber and then is conveyed to the product recovery step, where a cyclone or filter system separates the particles from the air. The cyclone uses centrifugal force to efficiently collect the dry powder, while additional filters capture any remaining fine particles.

**Fig. 1:**
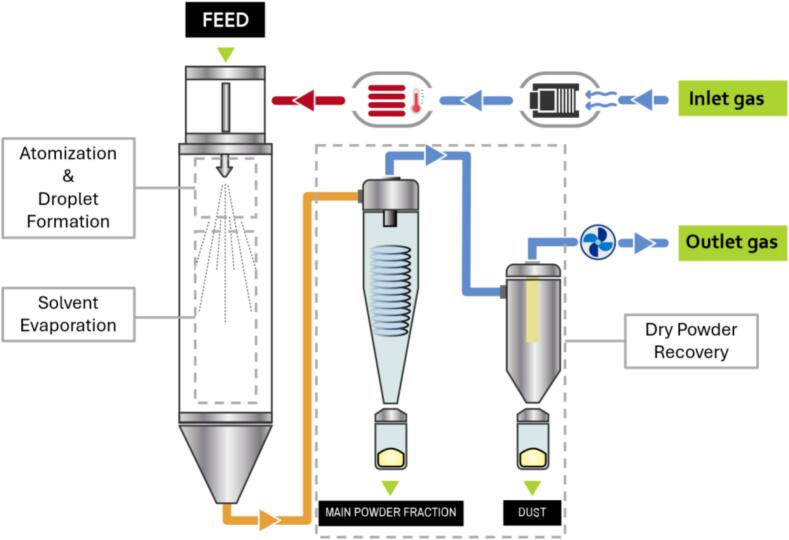
Spray drying process stages.

The characteristics of the final dried product are highly dependent on the drying rate, which is influenced by various process parameters (Sosnik and Seremeta, 2016; Qadri et al., 2023; Höhne and Gaukel, 2024). Summarizing the process description, it is clear that in spray drying, different phases follow one another in a continuous process that is usually divided and studied into three stages (Fig. 1):

1. Atomization and droplet formation.
2. Solvent evaporation.
3. Dry powder recovery.

Each phase is associated with critical issues that become challenges to guarantee the quality of the final product (Table 1).

**Table 1:** Spray drying process phases and key challenges.

**Process Phases**	**Procees Key Challenges**	**Product Quality Attributes**
**Atomization**	High stress at the gas-liquid interface Non-uniform droplet-size distribution Nozzle clogging	Potential protein denaturation or aggregation due to interfacial stress Broad particle size distribution leading to variability in flowability and dissolution Impairment of the integrity and activity of sensitive compounds by overheating near the nozzle
**Solvent evaporation/drying**	Thermal stress Controlling particle morphology Controlling residual humidity	Possible degradation and denaturation of thermolabile compounds Influence of bulk density caused by irregular particle shape
**Powder Recovery**	Low collection efficiency and yield loss Physical stress in the cyclone Variation in product quality	Reduction of process yield Change in size and surface properties due to particle breakage or friction Inconsistent product performance and stability between batches

### 2.1 Atomization and droplet formation

Atomization is the breaking of a liquid stream into an aerosol of individual droplets. This process can be driven by kinetic, centrifugal, pressure, or sonic forces, depending on the atomizer design. The atomizer selection is one of the main choices when considering spray drying process conditions: this component is responsible for the optimal spray of the product, which leads to optimal evaporation conditions and thus a finely dried powder (Masters, 2002; Cal and Sollohub, 2010; O'Sullivan et al., 2019).

#### 2.1.1 Rotary atomizers

**Fig. 2:**
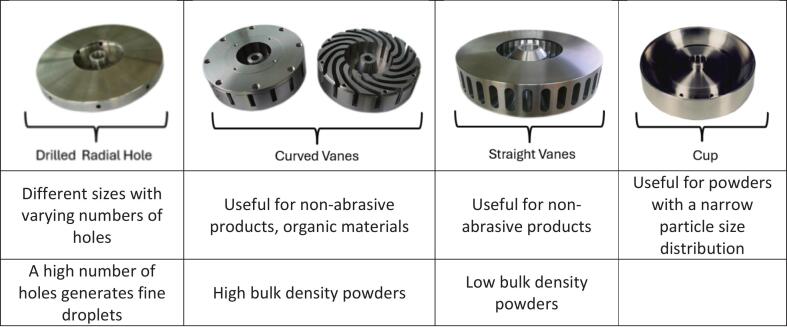
Different geometries of rotary wheels (reproduced from CMT Atomizers S.r.l., ITA, with permission).

The rotary atomizer utilizes centrifugal energy: due to the high rotational speed of the wheel, the liquid feed is propelled from the center to the outer edge of the disc, where centrifugal force accelerates it outward, breaking the liquid into fine droplets (Teske et al., 2001). There are very different designs of wheels on the market (Fig. 2), which, along with varying feeding rates and wheel velocities, can generate a wide range of droplet dimensions and size distributions suitable for very different products.

It is also possible to have wheels with inserts usually designed for atomizing abrasive products. The inserts protect the wheel from excessive abrasion-related wear. For heavy-duty applications, the wheels are also equipped with wear plates. Depending on the wheel size, a varying number of inserts can be arranged in single or double rows to provide additional durability (Ashgriz, 2011).

The mechanics of the rotary play a fundamental role in the final particle size distribution of the product; indeed, the wheel must be stable and avoid vibration, and it must be capable of high rotating velocities and thus high peripheral speed. In addition, the polishing of the vanes must be homogeneous and facilitate complete wetting of the internal surface (Masters and Vestergaard, 1975). The rotational speed of the wheel can typically range from 60,000 rpm for small pilot-scale setups with a wheel diameter of 50 mm to 14,000 rpm for large industrial-scale nozzles with a wheel diameter of 250 mm. The atomization performance of a rotating wheel is also dependent on the viscosity and surface tension of the liquid, the inertia of the liquid at the edge of the wheel, and the air friction between the liquid and the air interface.

At higher velocities, droplet formation occurs through the breakage of the liquid at the edge of the wheel, disrupting the liquid film emerging from the vane of the wheel. The higher the viscosity and surface tension of the liquid, the greater the uniformity of droplet sizes, resulting in a narrower particle size distribution, although this may yield larger mean droplet sizes.

Thanks to their mechanics, they can spray a variety of materials with different loads and viscosities, are easy to operate, reliable, and rarely affected by clogging. They are thus appreciated on an industrial scale; at the same time, they cannot be used in bench-top laboratory-scale spray dryers, because their characteristic “umbrella” spray pattern requires a minimum diameter and volume to be effective and to yield a dry product with high yields. For this reason, the minimum spray dryer size on which they are applied has an evaporative capacity of 5 kg/h.

#### 2.1.2 Pressure nozzles

**Fig. 3:**
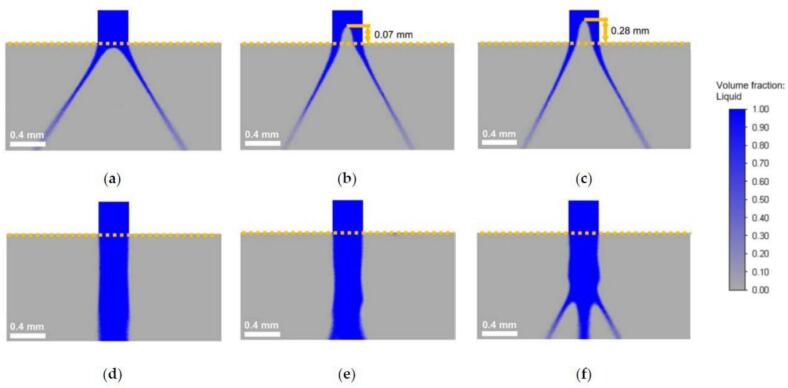
Instantaneous liquid fraction profiles during atomization with a pressure nozzle at varying viscosities and pressures. Panels (a–c) show the liquid flow at a viscosity of 10 mPa*s and pressures of 5 MPa, 10 MPa, and 20 MPa, respectively. At this lower viscosity, a partial air core forms inside the nozzle, aiding in the creation of a spray cone at the outlet. Increasing the pressure reduces the spray angle due to higher flow rates and a more defined air core. Panels (d–f) depict flow at a higher viscosity of 35 mPa*s under the same pressures. In these cases, no air core forms, and the liquid exits as a jet, which breaks into a spray cone further downstream. Higher pressures cause the jet to disintegrate more rapidly, but the absence of an air core leads to delayed atomization compared to lower-viscosity conditions (reproduced from Martinez and Gaukel, 2023, with permission).

Their working principle involves using liquid pressure energy to produce thin sheets measuring a few microns in thickness, which break into small droplets due to air friction and instability. These thin sheets are created at high speed and are very short, making them almost impossible to see with the naked eye due to the combination of high speed and turbulence at the nozzle. Even in this kind of nozzle, the most commonly used approach to produce thin liquid films is centrifugal force, which occurs inside the nozzle without mechanical parts in motion: the high pressure of the liquid is transformed by the peculiar swirl chamber geometry inside the nozzle into centrifugal force, and then the liquid is forced through a small orifice. To produce the centrifugal effect inside the nozzle, the swirl chambers usually impart a rotation that generates a typical hollow-cone pattern with an air core at the center (Fig. 3).

One of the main concerns of these nozzles is the wear of the swirl chamber and the orifice, which requires strict maintenance routines, frequent inspections, and the use of wear-resistant coatings or inserts (like tungsten carbide) to ensure the nozzles' longevity and to prevent any risk of particle contamination in the final product. Another significant drawback, particularly when processing suspensions, is the risk of clogging due to the small orifices. This can lead to operational disruptions and require frequent cleaning or maintenance.

Despite these challenges, pressure nozzles remain highly valued for their compact design and ability to produce a narrow droplet-size distribution, provided that appropriate maintenance and material choices are in place to mitigate wear and clogging (Ashgriz, 2011). They are employed only in pilot- and industrial-scale production because they require a significant amount of space: to accommodate their operational needs, the drying chamber must be sufficiently large, particularly in height. Therefore, generating a fully developed cone within a height of less than 5 m is challenging. In general, this type of atomizer could be inappropriate for treating protein-based compounds, as elevated pressure can cause protein degradation.

#### 2.1.3 Two-fluid nozzles

Two-fluid nozzles are the most versatile and widely used, particularly in laboratory settings, as they do not face the space constraints that pressure and rotary atomizers do. Furthermore, they are simple, scalable, cost-effective, easy to operate, and can be designed to meet hygienic standards, making them easy to clean and suitable for aseptic environments.

The atomization of the liquid into fine droplets occurs due to the kinetic energy of the gas, which generates significant frictional forces on the liquid's surface: the presence of high-velocity gas induces instability that first creates a rough break in the fluid and then gradually divides and breaks it into increasingly smaller droplets until it is completely atomized (Kemp et al., 2016). In this process, the velocity of the flow and the properties of the liquid (like viscosity and surface tension) play a leading role. A high relative velocity between the liquid and air is necessary for effective atomization: airspeed can approach sonic levels before contacting the liquid, and either flows in thin sheets or is rotated within the nozzle, depending on the design (Kim and Marshall, 1971). At small liquid feed rates, the air velocity alone can atomize the liquid, forming a fine spray. However, at higher feed rates, air cannot penetrate the thicker liquid as readily, leading to incomplete atomization and a wide droplet-size distribution. In such cases, a solid jet of liquid may form in the center of the spray, which is inefficient. For this reason, scaling up this type of nozzle may be challenging, and one of the widely used approaches is to introduce multiple nozzle heads to split the amount of liquid to be atomized into multiple atomizing sub-units.

**Fig. 4:**
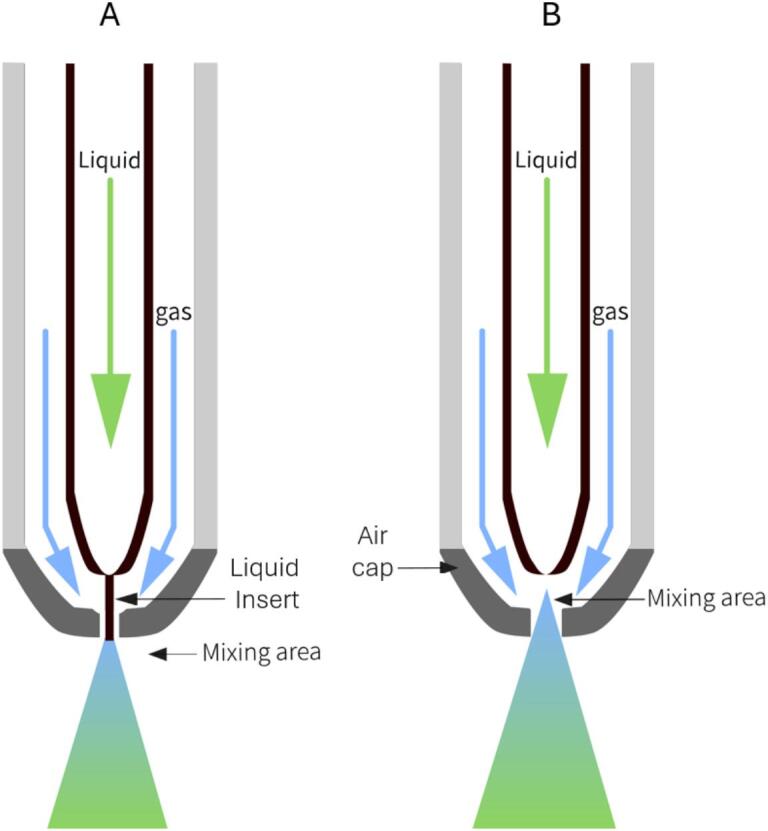
Two-fluid nozzle scheme. A: External Mixing, B: Internal Mixing.

This type of atomizing nozzle is composed of two distinct converging parts: the liquid insert and the air cap (Fig. 4). The liquid insert can be distinguished by its bore size, and it may have a different shape of the liquid duct and the outer surface. Moreover, the insert is usually carved to create channels for air to pass through when coupled with the air cap. These features make this a high-precision part (especially for small-size nozzles), which is critical for designing and producing to ensure correct spray cone formation and the functioning of the nozzle. An additional assembly variant often present in these nozzles is the mixing type: mixing between the two fluids, liquid and gas, can occur outside the nozzle cap or within it using a mixing chamber positioned between the liquid insert and the cap (Fig. 4). In internal mixing, air and liquid mix within the nozzle body before exiting, which is generally more energy-efficient because it requires less compressed air to achieve atomization. Internal-mixing nozzles also tend to produce a narrower droplet-size distribution, offering better control over particle uniformity. However, they are more prone to clogging when handling viscous liquids or those with suspended solids. In contrast, external mixing occurs outside the nozzle, with air and liquid exiting separately before combining. While this method consumes more energy and can result in a broader droplet-size distribution, it is more versatile and better suited for high-viscosity fluids or abrasive mixtures, as it reduces the risk of clogging. Therefore, internal mixing is preferable for fine, energy-efficient atomization with low-viscosity liquid solutions, whereas external mixing provides greater flexibility in handling diverse fluid properties.

The spray angle of these nozzles is generally narrow, which is better suited to towers with an elongated profile that develops in height rather than in width, as with rotary nozzles. The spray angle is usually around 30°-50° but may be increased to 80° for high-feed-rate nozzles and narrower to 10°, for the small laboratory-scale spray dryer, where the tower diameter could also be 15 cm (Huang et al., 2018). The atomization angle can be varied by adjusting the spray parameters: increasing in pressure at a constant feed rate produces a narrower cone; on the contrary, diminishing the feed rate of the product while maintaining the same gas pressure increases the spray angle (Kramm et al., 2023). A higher feed/gas ratio results in a larger cone in internal-mixing nozzle configurations. However, the spray angle disperses quickly and is challenging to maintain over long distances due to several factors: i) the interaction between the liquid and atomizing gas causes rapid turbulence atomization, resulting in droplet formation that begins to disperse immediately after exiting the nozzle; ii) air resistance further decelerates the droplets, leading to lateral expansion of the spray; iii) even the kinetic energy of the droplets decreases with distance, reducing the ability to control the spray angle. In addition, iv) turbulence and vortices generated in the process exacerbate the dispersion, making it difficult to sustain a consistent spray pattern over an extended distance.

In the two-fluid nozzles, the mouldability of air amount and pressure, along with fine adjustment of the air/fluid ratio, provides great versatility in droplet dimension control.

They are thus capable of producing a wide range of droplet sizes, offering greater flexibility than other atomizers. For example, unlike rotary atomizers, they can produce very fine particles and are therefore used to produce powder for pulmonary delivery (Kemp et al., 2013). Also, with these nozzles, the feed influences atomization: viscous materials generate a more dispersed droplet-size distribution, and the final product can be inhomogeneous (Camacho-Lie et al., 2023). Fluid viscosity, on the other hand, tends to increase droplet size by resisting atomization. Moreover, the droplet size is also influenced by the air-to-liquid mass ratio: high ratios generally reduce droplet size, though the effect diminishes beyond a ratio of 10:1, because over this limit, energy is expended without further significant change in droplet size. Additionally, increasing the relative velocity between the air and liquid enhances atomization, resulting in smaller droplets. Lastly, air density also impacts droplet size: higher air density reduces the size due to increased dynamic force (Cotabarren et al., 2018). These variables must therefore be carefully controlled to achieve optimal spray characteristics.

**Fig. 5:**
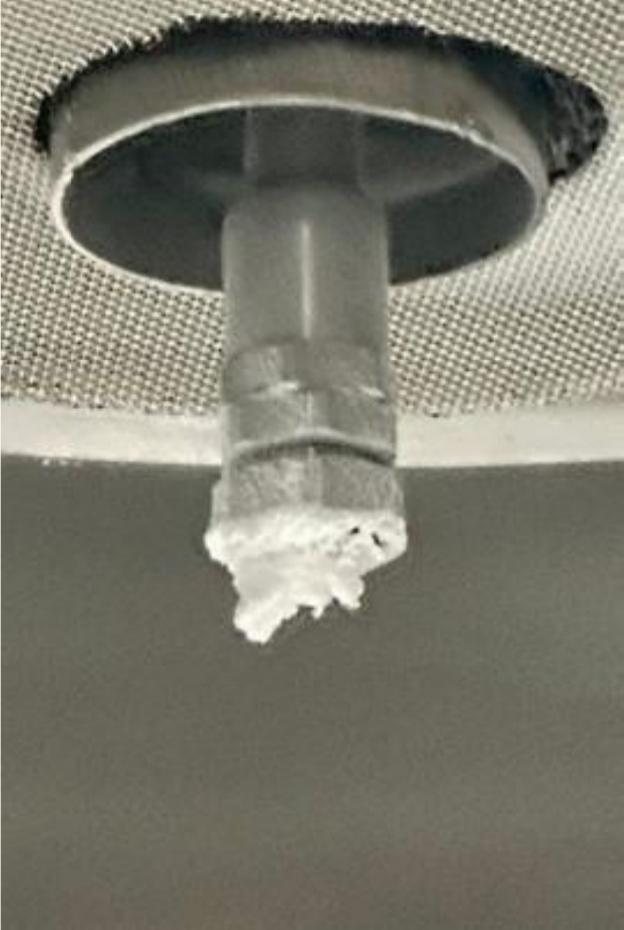
Deposition on top of the nozzle during the process.

The clogging may also occur in the two-fluid nozzle, particularly with small bore sizes or gelling materials that react to high temperatures, such as some polymers, starches, or gelatin-based solutions, which can thicken or solidify upon heating and lead to nozzle blockages, compromising spray quality and efficiency. Another typical issue may gradually occur during operation, particularly when processing fluids containing solids or substances prone to deposition: a crust may appear near the exit of the liquid insert. This phenomenon arises when, at the nozzle outlet, the fluid begins to form residues on the external surfaces due to partial evaporation or solidification. Over time, this deposit progressively obstructs the passages and compromises the nozzle's proper function (Fig. 5). As a result, the spray becomes asymmetric and inefficient, as the air and liquid are no longer evenly distributed. This leads to reduced atomization efficiency and deterioration in overall performance, necessitating cleaning or maintenance to restore regular operation.

#### 2.1.4 Ultrasonic atomizers

**Fig. 6:**
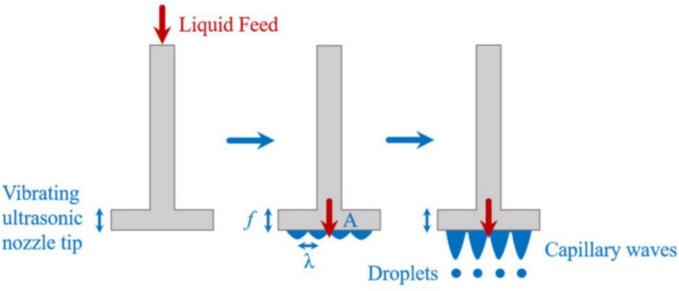
Droplet formation by transducer-and-horn design ultrasonic atomizer (sonotrode). The liquid forms a thin film over the resonant surface of the ultrasonic nozzle. Vibrations induced by the piezoelectric element generate capillary waves, leading to the detachment of droplets when the wave amplitude exceeds the liquid surface tension. The droplet size is primarily determined by the ultrasonic frequency, with higher frequencies producing smaller droplets (reproduced from Camacho-Lie et al., 2023 with permission).

These atomizers use sonic energy to break the fluid into droplets (Khaire and Gogate, 2020). This ultrasonic atomization operates by utilizing mechanical vibrations generated by a piezoelectric element to atomize liquid without the need for pressure or compressed air, generating a typical diffuse and soft mist: the vibrations transfer energy to the liquid, forming capillary waves on its surface; when enough energy accumulates, these waves become unstable and the liquid breaks into fine droplets. The piezoceramic element, which converts electrical energy into mechanical vibrations, is the core component of these nozzles. Many designs also include a wave amplifier to enhance the atomization process while reducing energy consumption. Ultrasonic nozzles can be classified by the method used to generate capillary waves, such as transducer-and-horn designs (standing waves) or vibrating capillary designs. In standing wave designs, piezoelectric transducers generate oscillations that create resonant waves, ultimately breaking the liquid stream into droplets (Fig. 6). In vibrating capillary designs, surface tension forces are manipulated by vibrations to eject droplets one by one from the fluid stream.

Despite limitations in flow rate and liquid properties, ultrasonic nozzles are appreciated for their precision in producing fine droplets with high consistency and uniformity. This makes them ideal for applications requiring a high degree of control, which is why they are predominantly used in the biomedical and electronics industries for coating applications. These coatings can slowly release therapeutic drugs (e.g., anti-inflammatory or antiproliferative agents), thereby preventing complications such as restenosis (McDermott et al., 2015). They are also used to apply biocompatible coatings, such as hydroxyapatite, to metal implants, such as joint replacements and dental implants, to promote bone integration and enhance biocompatibility by mimicking the mineral content of bone (Rios-Pimentel et al., 2023). Another interesting characteristic of this atomization method is the low traveling velocity of the produced droplets, which can lead to a more controlled, uniform drying process and a reduction in the risk of forming larger, undesired agglomerates that can occur with high-velocity sprays. Nevertheless, these nozzles have limited maximum flow rates because, as the flow rate increases, more vibration energy is required to break the liquid into fine droplets. Therefore, when the vibration energy reaches its maximum and cannot be further increased by increasing the frequency (typically set between 20 and 180 kHz), it may not be sufficient to effectively atomize a high-volume liquid stream, resulting in decreased atomization efficiency.

Ultrasonic nozzles are sensitive to liquid properties, such as viscosity, surface tension, and density, which can significantly affect atomization efficiency. High-viscosity fluids or those with high surface tension require more energy to form capillary waves, often exceeding the nozzle's capacity, leading to incomplete atomization or larger droplet sizes. Similarly, high-density liquids pose challenges because more energy is required to break them into fine droplets. These sensitivities limit the range of materials that can be effectively processed by ultrasonic nozzles, making them more suitable for low-viscosity, low-surface-tension, and low-density fluids (Ashgriz, 2011). Moreover, during atomization, this type of atomizer generates heat, increasing the inlet temperature of the drying gas and potentially degrading heat-sensitive compounds, such as protein-based ones (Ziaee et al., 2020).

While ultrasonic nozzles generally perform well with solutions, they may be prone to clogging when processing materials that can gel or form solid deposits, leading to interruptions in atomization and reduced efficiency, especially if solids are present in the feed fluid.

**Fig. 7:**
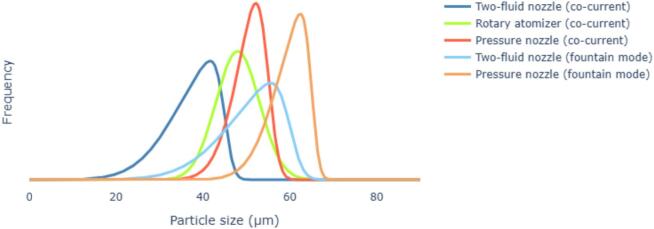
Example of how a comparison of particle size distributions of different nozzles may appear.

Fig. 7 exemplifies the differences in PSD generated by the various types of nozzles: each type produces a peculiar distribution curve that reflects its characteristic atomization and droplet generation. For instance, two-fluid nozzles typically produce a broader, more varied droplet size distribution, resulting in a wider particle distribution. In contrast, rotary atomizers typically produce a more uniform droplet size, resulting in a narrower distribution curve and a smaller mean particle size. Pressure nozzles, on the other hand, generally yield larger droplets, leading to a PSD shifted to the right. Additionally, nozzles operating in fountain mode tend to produce larger particles with broader distributions, as the upward redirection of the spray introduces greater variability. Overall, the shape of each distribution curve provides insights into the droplet formation process, which directly influences the Sauter Mean Diameter and the consistency of the final powder product (Camacho-Lie et al., 2023).

The spray nozzle can also determine the speed and path that the particles follow as they travel. Not only are there differences between the type of atomization cone produced and its angle (as described above for the various types of nozzles), but with some of these nozzles, it is also possible to use counter-current spray or fountain mode spray, which significantly changes the residence time, the path, and the interaction between the droplets. In a co-current system, both the hot drying air and the droplets move in the same downward direction; this allows for a gentler drying process, where the hottest air contacts the wettest droplets, gradually cooling as the particles dry, making it ideal for heat-sensitive materials. Instead, in a counter-current system, the hot air flows against the direction of the droplets, creating more intense drying conditions. Here, the driest particles meet the hottest air, resulting in a shorter drying time but potentially higher thermal stress on the material. This configuration is suited for products that can withstand higher temperatures and require rapid drying. For counter-current spray, only two fluid nozzles and pressure nozzles are used.

**Fig. 8:**
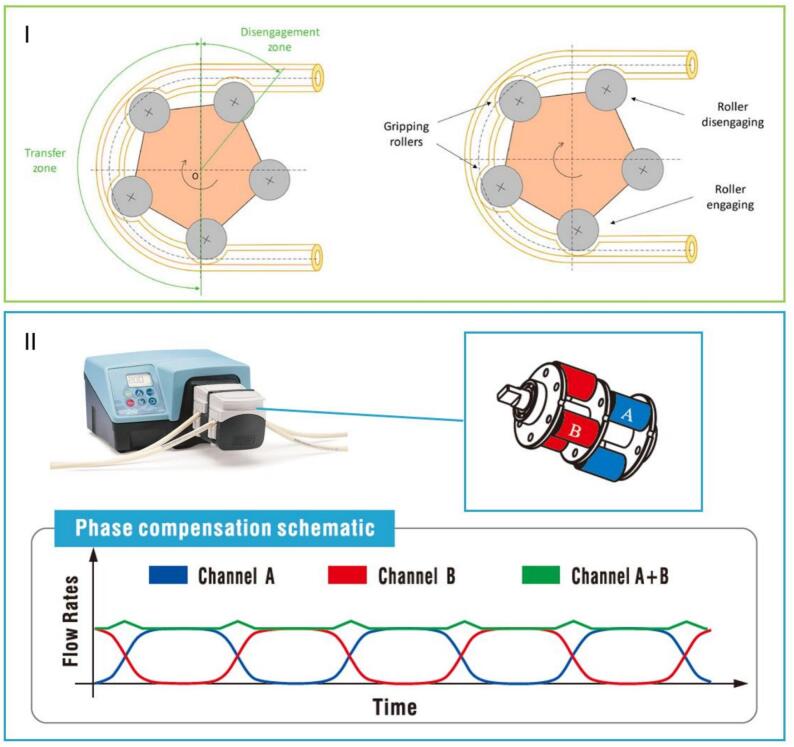
I: Peristaltic pump functioning. II: Single and Asymmetrical double head rollers comparison (Adapted from Ferretti et al., 2023 with permission).

Pumping is a step upstream and is often considered secondary to the atomizer. However, it plays a crucial role in spray drying by ensuring a consistent liquid feed to the atomizer. Peristaltic pumps are common as they offer precise control over flow rate and minimize contamination through their closed-loop system (Formato et al., 2019). However, one drawback of peristaltic pumps is the inherent pulsation in the flow, which can lead to variability in the atomization process. This discontinuous delivery of the feed can result in fluctuations in droplet size, ultimately increasing the variability of the final powder product's particle size distribution and other key properties (Deiringer et al., 2023). Different types of pumps, such as gear or diaphragm pumps, are also used in spray drying, but each presents its own challenges regarding flow consistency. A steady, continuous flow is essential for achieving uniform droplet formation, reducing variability, and producing a more consistent final product. To overcome pulsation in peristaltic pumps, particularly at low flow rates, special double-head models with asymmetrical rollers are often used (Fig. 8). The increased number of rollers, along with their asymmetric disposition, helps to reduce the pulsation effect by alternating the flow between two pump heads, creating a more continuous and steady delivery of the liquid and avoiding the “pulsation dead zone” effect that happens during the brief interval where no roller is compressing the tube. This design also allows the pump to be positioned farther from the atomizer, which can be beneficial in specific setups where operational or space constraints necessitate a greater distance between the pump and the atomizer (Ferretti et al., 2023).

### 2.2 Solvent evaporation

After atomization and during the drying step of the process, the first critical condition is to create a flow pattern within the tower that effectively prevents wall deposition and ensures complete droplet drying (Kieviet et al., 1997).

As regards wall deposition, it may be strictly related to the drying and evaporation phase: if the time or temperature is insufficient to ensure the drying of the droplet and the complete solvent evaporation during the traveling of the particle, this may result in the product sticking to the wall, and possible build-up of material during the process (Fletcher et al., 2006). On the other hand, wall deposition may be caused by local turbulence and vortices generated by the nozzle or by an inadequate airflow. These phenomena may remain confined to critical areas, generating convecting flows that increase the particle's residence time in the hot region of the tower or cause it to ascend into lower-pressure regions. They may also cause deposition in areas near the nozzle or even within the air entrance, leading to browning and burning of the particles. In this step, product characteristics also play a role. Specifically, the droplet size, density, and even shape can cause different behaviors, altering the trajectory and the amount of air friction that dissipates the significant amount of energy used in atomization. Even though the initial droplet speed is often much higher than the drying air inlet velocity, the rapid deceleration caused by air frictional forces acting on the droplet surface ensures that wall deposits in industrial-scale spray dryers are rarely due to the momentum of the droplets. Instead, with good and complete atomization, wall deposits are typically caused by other factors rather than the “wall painting” effect (Sundararajan et al., 2023).

When the droplets lose their kinetic energy, their path becomes predominantly governed by the airflow dynamics within the drying chamber. The droplet trajectory and behavior can be studied using Computational Fluid Dynamics (CFD) models (Camacho-Lie et al., 2023). These simulations are a powerful tool that includes information on trajectories and interactions with the drying air, allowing the modeling of intricate dynamics of airflow, droplet dispersion, and heat and mass transfer as droplets undergo atomization and phase change. However, accurately simulating these conditions poses significant challenges due to the complexity of the spray-drying environment. First, the atomization process involves a highly turbulent, multi-phase flow, where the transition from the liquid to the vapor phase must be captured in real time. This requires detailed modelling of droplet breakup, coalescence, and evaporation, which are influenced by droplet size, speed, and distribution. Additionally, the introduction of hot air further complicates the simulation, as heat transfer and resulting temperature gradients significantly affect evaporation rate and overall drying kinetics. While CFD can provide valuable insights into droplet trajectories and the interaction between droplets and air, accurately predicting these phenomena requires substantial computational power and advanced modelling approaches to account for the numerous variables involved in such a complex system. For these reasons, the models are often simplified to assume the droplet mass and shape are constant along their path through the spray dryer. Furthermore, not all droplets are included in the simulation due to its high computational complexity; instead, only a few representative spheres or droplets are simulated to approximate the behavior of the entire system. In some cases, for simplicity, the simulation may focus solely on the airflow trajectory, without considering droplets, especially when the primary goal is to understand the dynamics rather than the detailed interactions between the air and droplets.

As regards the evaporation of the solvent and the consequent drying of the droplets, this process can be simplified into distinct phases of temperature and drying rate (de Souza Lima et al., 2020). Initially, when the droplet enters the drying chamber, it begins to absorb heat from the surrounding hot air. At this stage, the energy is primarily used to raise the droplet's temperature, resulting in minimal evaporation and a relatively low drying rate. As the droplet reaches a critical temperature, typically close to the wet-bulb temperature, evaporation begins in earnest. This marks the start of the constant-rate drying period (Liang et al., 2021), during which moisture is rapidly removed from the droplet at a steady rate. During this phase, the droplet maintains a relatively stable temperature, as the energy supplied primarily evaporates surface moisture. This ensures the product is not exposed to high temperatures early in the drying process, which is especially beneficial for heat-sensitive materials, helping preserve their integrity and prevent thermal degradation. This steady-state condition ensures that the drying process occurs in a controlled manner. By maintaining a stable temperature, this phase effectively protects the heat-sensitive compound from the adverse effects of elevated heat exposure that could otherwise lead to structural damage or functional deterioration. Moreover, this controlled evaporation process helps preserve the material's integrity and quality, preventing thermal degradation that could compromise its desired properties or efficacy. Overall, the constant-rate drying period plays a vital role in ensuring a safe, efficient, and gentle drying process that safeguards the material's original characteristics and performance.

Additionally, this phase enables rapid moisture removal, as the bulk of the liquid content evaporates quickly, leading to a significant reduction in droplet size. As drying continues, the droplet forms a solid outer layer. This marks the transition to the falling-rate drying period, where the drying rate declines sharply. At this point, the remaining moisture is trapped within the particle's solidified matrix and must diffuse through the solid structure to evaporate. Consequently, the drying rate slows considerably, and the droplet's temperature rises, as less energy is required for evaporation and more is directed toward heating the solidified particle. The drying rate in this phase is influenced by both the internal diffusion of water and the crust's permeability. While this can introduce challenges, such as the potential for internal stress or particle cracking, it also offers greater control over the final particle structure and stability.

During these two phases, the material loses moisture, which can be categorized into bound and unbound moisture, and which behaves differently during the drying process. Unbound moisture is water free to evaporate from the surface of the material. It exists in non-hygroscopic materials and does not interact chemically or physically with the solid matrix. Unbound moisture is typically removed first during the constant-rate period, when evaporation is rapid and driven by a high moisture gradient. Bound moisture is instead tightly held within the solid matrix and, as such, requires more energy to be removed; it is associated with hygroscopic materials. Bound moisture exerts an equilibrium vapor pressure lower than that of unbound water at the same temperature, making it harder to evaporate. It is typically removed during the falling-rate period of the drying process, when the rate of unbound moisture removal decreases. Drying stops when the remaining moisture in the material is in equilibrium with the surrounding air's relative humidity. Once this point is reached, no further drying occurs unless conditions change (e.g., increasing the temperature or decreasing the humidity).

**Fig. 9:**
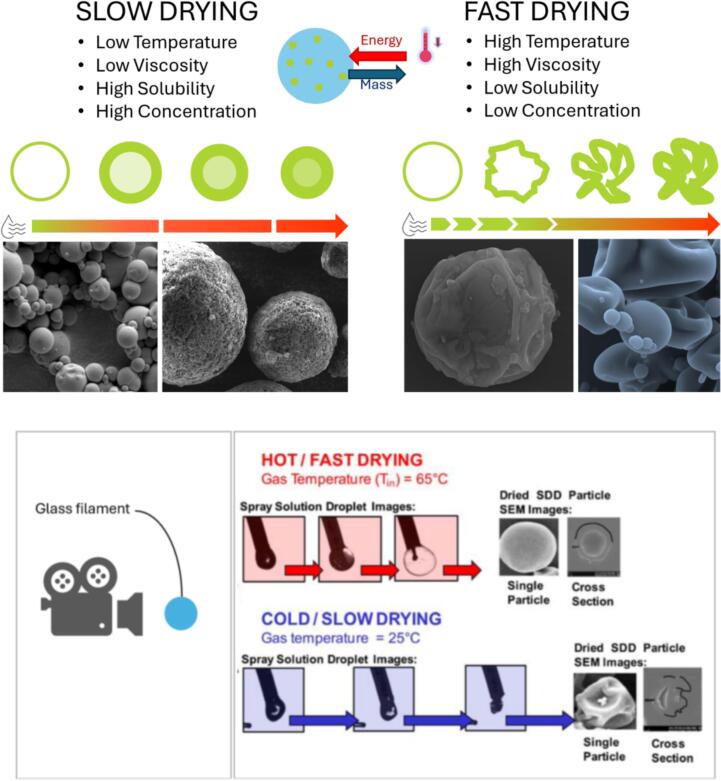
Different particle morphologies obtainable with slow drying kinetics and fast drying kinetics and an example of SDD glass filament images taken with a high-speed camera of hot/fast drying conditions and cold/slow drying conditions when a film-forming polymer is used in acetone solution (adapted from Dobry et al., 2009 with permission).

The gradual transition from a liquid droplet to a solid particle, along with changes in heat transfer and evaporation dynamics, plays a critical role in determining the final characteristics of the dried powder, like particle size, structure, and residual moisture content (Eijkelboom et al., 2024). For example, the drying kinetics (fast or slow) can influence the particle morphology (Malafronte et al., 2015): slow drying, guided by factors such as low temperature, low viscosity of the slurry, and high solubility and concentration of the dispersed product, can lead to a dense and spherical particle. A high temperature, combined with high viscosity, low solubility, and concentration, results in a hollow particle that can inflate, collapse, or shrink (Fig. 9).

Overall, understanding and optimizing these phases with different components and formulations allows for more efficient spray drying while producing high-quality powders with consistent particle sizes and stable active ingredients, and it stands at the basis of particle engineering that can be achieved thanks to the spray drying process (Masters and Vestergaard, 1975; Boel et al., 2020). In detail, the relationship between convective evaporation at the droplet's surface and the diffusive migration of moisture within the droplet can be quantitatively captured by the Péclet number (*Pe*) (Lintingre et al., 2016):

Pe=Rν/D

where *R* is the droplet radius, *ν* is the evaporation velocity, and *D* is the diffusion coefficient of moisture within the droplet.

This dimensionless parameter provides critical insight into the rate of crust formation and, consequently, the morphology of the dried particle (Fig. 9). Understanding and controlling the Péclet number is essential for optimizing the drying process and achieving the desired particle properties. A high Péclet number (*Pe* > 1) indicates that convective transport dominates, meaning the liquid near the droplet surface is evaporating faster than it can be replenished from the droplet interior. This leads to rapid crust formation and can result in particles with hollow or porous structures. This effect is often observed in processes where fast evaporation is desirable to prevent solute migration and preserve the stability of heat-sensitive compounds. On the other hand, a low Péclet number (*Pe* < 1) implies that diffusive transport dominates, allowing the moisture to gradually migrate from the interior of the droplet to the surface. In such conditions, evaporation occurs more uniformly throughout the droplet, resulting in denser, more compact particles.

For applications where particle density and mechanical strength are critical, controlling the Péclet number is therefore essential. By adjusting process parameters such as feed concentration, droplet size, and air temperature, the Péclet number can be tuned to control the final particle morphology (Table 2). This makes the Péclet number a crucial tool for optimizing the spray drying process for specific applications (Lechanteur and Evrard, 2020).

**Table 2:** Comparison of fast drying (*Pe* > 1) and slow drying (*Pe* < 1) impact on particles' properties.

**Properties**	***Pe* > 1**	***Pe* < 1**
Morphology	Shrivelled	Spherical
Bulk Density	Low	High
Residual Solvent	Low	High
Compressibility	High	Low
Particle Structure	Contracted/Inflated^a^	Packed

a Breakage may occur.

In recent years, significant advances have been made in studying drying kinetics and particle formation in spray drying processes, even by leveraging Single Droplet Drying (SDD) techniques (Eijkelboom et al., 2023). These approaches provide insight into particle formation from droplet-scale up to pilot-scale operations, bridging fundamental research and industrial applications. The advantage of SDD lies in its ability to simplify and closely monitor the drying process at the microscopic level, offering crucial data on drying rates, morphological evolution, and phase transitions during droplet evaporation. By studying individual droplets under controlled conditions, researchers can replicate and predict how particles will behave during large-scale spray drying operations. This is particularly useful when optimizing spray drying for pharmaceuticals or food products, where the final particle structure (e.g., size, porosity, and agglomeration) plays a pivotal role in product quality (Ehring, 2008).

SDD can be performed through various techniques, such as acoustic or aerodynamic levitation or using a free-fall method. Each of these techniques allows for close monitoring of drying kinetics without interference from external forces that are typically present in large-scale spray dryers (Dobry et al., 2009). During the initial constant-rate phase, evaporation is primarily limited by external heat and mass transfer. In contrast, in the falling-rate period, internal mass transfer within the droplet becomes the limiting factor. This progression is crucial in understanding how crust formation affects the final particle morphology and the residual moisture content. As the droplet dries, mechanical stresses can form in the crust, affecting both the particle's internal structure and surface texture, especially when crust formation precedes complete moisture removal (Boel et al., 2020; Eijkelboom et al., 2023). The ability to study such processes with SDD enables more precise control over variables such as droplet size, solute concentration, and drying temperature. This, in turn, allows for fine-tuning of particle characteristics to meet specific needs, such as improving flowability, enhancing reconstitution properties, or adjusting bulk density in spray-dried powders. Despite its benefits, SDD does not fully replicate the complexity of industrial spray drying conditions. For instance, droplet-droplet and droplet-wall interactions, which are prevalent in large-scale systems, are absent in SDD. Moreover, droplets in SDD experiments tend to be larger, and drying times are longer than in industrial applications. As a result, while SDD provides valuable insights into particle formation and drying behavior, translating these findings to full-scale operations requires additional research and pilot-scale trials (Vicente et al., 2013; Boel et al., 2020).

### 2.3 Dry powder recovery

The recovery of dried powder from spray drying operations is a critical final stage that requires careful consideration of both separation efficiency and collection methods: efficient recovery minimizes product loss and ensures compliance with environmental standards for air pollution.

The complete dedusting of process air is often achieved using a combination of separation systems, which can be classified into primary separators, which recover the bulk of the product directly from the drying process, and secondary separators, which collect finer particles from the exhaust air. The choice of equipment must balance factors like capital and operational costs, ease of maintenance, and space requirements (Graham et al., 2010; Gawali and Bhambere, 2015).

Cyclones are among the most common types of collection devices used as primary separators, offering high separation efficiency for larger particles. The reverse-flow cyclone device relies on the swirling motion of gas to create a double-vortex flow pattern, consisting of an outer vortex with downward axial flow and an inner vortex with upward axial flow. As the gas spirals downward in the cyclone's separation chamber, particles are pushed outward by centrifugal force, colliding with the walls and moving toward the dust exit. After entering the conical section, the gas is redirected inward and rises through the inner vortex, eventually exiting through the vortex finder. The cyclone's geometry is crucial to its performance, as each component affects the separation process (Singh and Rana, 2021). In addition to its geometric design, the pressure dynamics inside the cyclone are fundamental for efficient particle separation (Fatahian et al., 2021): as the gas spirals downward, energy losses from friction, particle collisions, and wall interactions cause the total pressure to drop; simultaneously, the static pressure decreases as the gas velocity increases near the walls, intensifying the centrifugal force on the particles and driving them outward for collection. This flow and pressure distribution ensure that particles are effectively separated, with the cleaned gas exiting through the central vortex (Wang et al., 2023). A greater total pressure drop indicates higher energy consumption, underscoring the importance of optimizing cyclone design to balance separation efficiency and operational costs. Moreover, the static pressure profile helps to determine where re-entrainment of particles might occur, particularly in areas where the inward-directed gas flow might pull already separated particles back into the stream (Shephered and Lapple, 1939; Pandey et al., 2022).

The interplay between centrifugal and drag forces can explain why cyclones are more challenging to collect fine particles than coarse ones. As particles enter the cyclone, they are subjected to an outward centrifugal force and an inward drag force. The centrifugal force is proportional to the particle mass, and thus the cube of the particle diameter (*x*^3^), meaning that larger particles are pushed outward more strongly. In contrast, the drag force acting on the particle is proportional to its diameter (*x*), according to Stokes' Law. This means that smaller particles, being lighter, experience less centrifugal force but are more affected by the inward drag force, making them more difficult to separate (Hoffmann et al., 2003). Therefore, while large particles are more easily separated and collected near the cyclone walls, fine particles often remain suspended in the gas stream, resulting in lower collection efficiency. This is why, for fine particle collection, additional secondary separators, such as bag filters or electrostatic precipitators, may be required to ensure efficient recovery. In the literature, some solutions to improve particle separation involve reducing the cyclone diameter, thereby increasing the tangential velocity inside the cyclone (Maury et al., 2005). This design change significantly increases centrifugal force, reducing the cut-off diameter for particle separation and enabling the system to trap finer particles more effectively. However, this benefit comes with certain drawbacks: the smaller diameter results in a higher pressure drop across the cyclone, leading to more restricted airflow and requiring greater energy to maintain process efficiency. This higher pressure drop also limits the amount of process air that can pass through the system, potentially reducing overall throughput and making the system less energy-efficient. Therefore, while a smaller cyclone provides better particle separation, particularly for fine particles, it does so at the expense of increased energy consumption and reduced process airflow, making it less economically favorable in terms of operational efficiency. Moreover, it is essential to note that a smaller cyclone tends to develop higher temperatures due to the kinetic energy of the air rapidly converted into heat in a smaller diameter and the increased frictional and tangential velocity, so the cyclone wall could heat up to a point where the product could become sticky, leading to deposits on the walls, which then reduces the powder yield if not appropriately managed (Bögelein and Lee, 2010).

In cyclone design, the pressure drop is primarily determined by the vortex finder rather than by components such as wall friction or inlet design. This is because the vortex finder serves as the primary pathway for the gas to exit the cyclone, and its dimensions and design create a bottleneck for the airflow (Wang et al., 2023). In contrast, wall friction has a lower impact on the pressure drop because it results in relatively minor energy losses compared to the significant changes in velocity and flow direction that occur near the vortex finder. Similarly, the inlet design contributes to the overall pressure drop, but its influence is less significant than that of the vortex finder, which exerts greater control over the gas flow exiting the cyclone. Thus, the vortex finder's geometry and size play the most critical role in determining the overall pressure drop in the cyclone system, making it the dominant factor in airflow restriction and energy losses. Different inlet configurations are used in cyclones; among them, the wrap-around inlet differs significantly from traditional circular and rectangular slot inlets (Hoffmann et al., 2003). Unlike the slot inlet, where the gas flow is compressed against the wall, leading to higher velocities and stronger centrifugal forces, the wrap-around inlet distributes the gas more uniformly across the cyclone's circumference. This results in a more stable flow pattern with fewer fluctuations and reduced wall wear, enhancing the cyclone's durability and long-term performance. In comparison, the rectangular slot inlet can generate higher tangential velocities, improving the separation of larger particles, but at the cost of increased wall erosion and the potential for particle re-entrainment due to uneven flow. The circular inlet, often considered a basic design, offers a balance between flow uniformity and velocity, but may not optimize either as effectively as the wrap-around or slot inlets (Hoffmann et al., 2003).

After the cyclone separator, the process filter blocks the particles that are not retained by the cyclone. In some cases, a wet scrubber is often used in conjunction with a cyclone and filter for enhanced particle removal. In a wet scrubber, droplets are introduced into the outgoing gas stream, either by spraying or by shearing a liquid source, forming droplets that interact with remaining dust particles. The dust particles collide with the droplets, becoming wet and heavy; as a result, the particle-laden droplets settle by gravity into a collection basin at the bottom of the scrubber, where they remain in suspension or dissolve in the liquid, effectively separating from the clean gas that exits the scrubber. Wet scrubbers, particularly Venturi scrubbers, are highly efficient at capturing fine particles, as the high-speed flow promotes effective coalescence between the particles and droplets. Additionally, the pressure drop in wet scrubbers remains relatively stable, and degradation of the scrubbing medium is usually not a significant issue. However, one drawback is that the particles and any water-soluble substances in the gas are transferred to the liquid phase, leading to a slurry that must be handled appropriately to prevent turning an air pollution problem into a water pollution problem.

## 3 Role of formulation components

Formulation design is a crucial step in developing a spray-dried product. The selection of excipients must be guided not only by the process requirements but also by the intended use of the final product, as not all excipients are suitable for every route of administration. If oral spray drying powders can be formulated quite freely, the excipients – for example, for inhalable, parenteral, and nasal spray drying powders – have to be selected carefully, as the list of FDA-approved excipients is often short. Despite this, in the research field, a wide range of materials is tested for various purposes. They may be incorporated into formulations based on their specific functions, such as facilitating handling or enhancing bioavailability. Fillers, mucoadhesives, preserving, stabilizing, and film-forming agents are the most common excipient categories considered.

In spray drying processes, the formulation plays a crucial role in influencing the atomization, the buildup on dryer walls, the recovery, and the final product characteristics like morphology, residual moisture, and flowability of the powder (Maa et al., 1997; Lowinger et al., 2015; Giovannelli et al., 2021; Ordoubadi et al., 2023; Vickovic et al., 2023; Barceló-Chong et al., 2024a). One of the key factors that leads to the stickiness of the product on the wall is the Glass transition Temperature (Tg), as materials approaching or exceeding their Tg undergo a transition from a solid state to a rubbery, sticky phase. Moisture content is the major contributor, as higher moisture levels lower Tg, increasing the likelihood of surface adhesion. Carbohydrates and proteins in the formulation can retain moisture, further promoting stickiness (Turchiuli et al., 2011), although fat content generally reduces stickiness by forming a protective layer that minimizes surface adhesion. On the contrary, excessive lipid content can lead to surface adherence issues. Therefore, careful design and control of the formulation and its components, such as balancing moisture, carbohydrate, protein, and fat content, are crucial for managing stickiness during spray drying and ensuring efficient production.

Excipients used in spray drying can be broadly categorized into several macro-groups, including sugars and polyols, polymers, proteins, lipids, and gums, each contributing unique properties to the formulation (Table 3).

**Table 3:** Relationship between excipient function and impact on the final spray-dried product.⁎

**Category**	**Function**	**Impact on Final Product**	**Examples**
Sugars & polyols	Bulking agents; stabilizers; moisture regulators; crystallizing agents; matrix formers	Improvement of protein stability; reduction of protein degradation; influence of particle morphology; enhancement of flowability	Trehalose, sucrose, mannitol, lactose
Polymers	Amorphous stabilizers; anti-sticking agents; film formers; release modifiers	Prevention of recrystallization; control of drug release rate; reduction of stickiness; enhancement of solubility and bioavailability	HPMC, HPMCAS, PVP, HPC
Amino acids & proteins	Stabilizers for protein-based drugs; surface modifiers; film formers	Improvement of particle aerosolization; prevention of protein-based drug aggregation and denaturation	Leucine, glycine, arginine, bovine serum albumin
Lipids	Moisture barriers; surface coatings; encapsulating agents	Enhancement of stability; reduction of stickiness; improvement of solubility of hydrophobic drugs; protection from degradation	Lecithin, phospholipids, triglycerides
Gums & polysaccharides	Emulsifiers; stabilizers; carriers; mucoadhesive and release-modifying agents	Enhancement of encapsulation efficiency; improvement of moisture retention; control of drug release profile; stabilization of drugs; prolongation of the residence time at the absorption site	Arabic gum, maltodextrin, chitosan, CMC, alginate

⁎ See Paragraph 3.2 for acronyms.

### 3.1 Sugars and polyols

Sugars are used as bulking agents and flow enhancers. When the active compounds are biopharmaceuticals, these excipients can also serve as protein stabilizers, protecting the protein from thermal stress and preventing degradation. Among these excipients, the use of lactose as a bulking agent in spray-dried formulations is limited, as it typically produces an amorphous product with high hygroscopicity and low stability. Furthermore, lactose exhibits reducing properties, making it incompatible with biopharmaceutical compounds such as peptides and proteins.

Otherwise, trehalose, sucrose, and mannitol are widely employed for their glass-forming abilities, providing amorphous stabilization of APIs, particularly in protein formulations (Mensink et al., 2017; Rajagopal et al., 2018; Dieplinger et al., 2023). Trehalose acts by forming hydrogen bonds with proteins, thereby preserving their native conformation and preventing aggregation during drying (Eedara et al., 2021). In addition, polyols such as mannitol are frequently used due to their crystallization behavior, which improves the physical stability of the dried product and reduces stickiness by promoting crystalline matrix formation (Arte et al., 2024). However, depending on the application, the choice between crystallizing (e.g., mannitol) and non-crystallizing sugars (e.g., trehalose) must be carefully considered based on the desired product characteristics. For example, in the case of protein-based compounds, the stability effect of these two excipients is related to their ability to interact at the molecular level with the protein (Wilson et al., 2019; Chen et al., 2021). Trehalose, with a high glass transition temperature, remains completely amorphous after spray drying, which is crucial because a homogeneous amorphous matrix reduces molecular mobility and prevents phase separation. Moreover, the formation of hydrogen bonds between trehalose and the protein replaces those with water, thereby helping to preserve the protein's native conformation. Unlike trehalose, the effect of mannitol is much more complex and less effective due to its marked tendency to crystallize, which causes a phase separation between the protein, which remains amorphous, and the crystalline excipient. This immiscibility reduces the protective interactions between the protein and the excipient, making the protein-based compound more susceptible to stress and degradation.

### 3.2 Polymers

Polymers like hydroxypropylmethylcellulose (HPMC), polyvinylpyrrolidone (PVP), and hydroxypropylmethylcellulose acetate succinate (HPMCAS) are essential in the formulation of ASDs (Friesen et al., 2008; Patel et al., 2013; Vodak and Morgen, 2014; Honick et al., 2020; Sauer et al., 2021). These excipients prevent drug recrystallization by stabilizing the drug's amorphous form and improving its solubility and bioavailability (Shah et al., 2014). Moreover, cellulose derivatives, such as HPC and HPMC, are widely used not only as anti-sticking agents in spray-dried formulations (Cheng et al., 2024) but also in the formulation of spray-dried powders due to their film-forming capabilities and ability to control drug release profiles. HPMCAS, in particular, is commonly used because it maintains the drug in a supersaturated state after dissolution, thereby enhancing its absorption (Shah et al., 2014). Additionally, polymers such as cellulose derivatives not only stabilize the amorphous state but also influence the Tg, thereby affecting the product's stickiness during processing. Higher polymer content typically increases Tg, reducing the risk of stickiness and improving the processability of spray-dried powders.

Polyvinylpyrrolidone (PVP) has been widely utilized due to its excellent solubility, stability, and compatibility with a broad range of biomolecules. In particular, it has been combined with other excipients, such as saccharides and amino acids, to develop vaccines against human papillomavirus (HPV) successfully (Kunda et al., 2019). These combinations have proven effective in stabilizing antigens and enhancing the overall formulation performance. Furthermore, highly porous particles useful as efficient pulmonary dosage forms can be produced when PVP is blended with polyethylene glycol (PEG).

### 3.3 Proteins and amino acids

Proteins, peptides, and amino acids are valuable excipients in spray drying powders, particularly in inhalable formulations, where particle size and dispersion are critical (Yue-Xing et al., 2018; Mah et al., 2019; Zhang et al., 2022). Amino acids are selected to act as stabilizing agents for protein-based drugs, even if their precise mechanism of action is not identified, and also because their presence improves particle aerosolization and favours the reconstitution of parenteral dosage forms (Pinto et al., 2021). Among the amino acids used in the formulation of spray-dried protein-based drugs, leucine, glycine, arginine, and histidine are the first choice. Leucine, for example, has a low molecular weight and surface-active properties that make it ideal for improving aerosolization in pulmonary delivery systems (Eedara et al., 2021). It is distributed on the particle surface, forming an external layer and improving the aerosol performance of the powder. In addition to their ability to form films and reduce surface tension, proteins can also act as stabilizers for APIs, especially in formulations containing biopharmaceuticals, by preventing aggregation and denaturation during drying. The most frequently used are bovine serum albumin, human albumin, and lactoglobulin. The presence of these compounds in the formulation promotes protein-protein interactions, preventing enzyme aggregation and unfolding and protecting enzymes from deactivation during spray drying (Yoshii et al., 2007).

### 3.4 Lipids

Lipids play a dual role in spray drying, influencing both the stability and stickiness of the formulation (Shetty et al., 2022; Corzo et al., 2023). These excipients are distributed on the surface of the spray-dried particles, forming a protective layer that limits moisture absorption and prevents the crystallization of the sugars present in the formulations.

Phospholipids and triglycerides are commonly used to encapsulate hydrophobic drugs, improving their solubility and protecting them from degradation. Lipids, such as lecithin, are also used to form protective coatings around particles, reducing their tendency to stick to the dryer walls during processing (Alhajj et al., 2021). However, the behavior of lipids must be carefully managed, as they can melt and adhere to hot surfaces, leading to fouling and process inefficiencies. Balancing lipid content and controlling drying temperatures is therefore critical.

### 3.5 Gums and polysaccharides

Gums such as arabic gum and polysaccharides, such as maltodextrin, are frequently used as carriers or stabilizers in spray drying, particularly in food and nutraceutical applications (Mishra et al., 2014; Arumugham et al., 2023; Vargas et al., 2024; Diana et al., 2025). Arabic gum is an excellent emulsifier and film-forming agent that protects sensitive bioactive compounds during spray drying, while maltodextrin helps control viscosity and improve particle stability (Eedara et al., 2021). Maltodextrin, due to its high molecular weight and good solubility, is often used to produce particles with improved flowability and rehydration properties. These excipients are particularly important in formulations that require moisture retention and controlled release, making them ideal for encapsulating flavors, probiotics, or sensitive bioactive compounds.

Maltodextrins offer an interesting combination of characteristics, providing amorphous stabilization while also mitigating stickiness in formulations with high sugar content. The degree of hydrolysis of maltodextrin, as measured by dextrose equivalent (DE), plays a crucial role in its performance during spray drying (Nadali et al., 2022). Lower DE maltodextrins are less hygroscopic, which improves process stability and reduces stickiness; however, they require higher drying temperatures and longer drying times, making them more challenging to process. On the other hand, higher DE maltodextrins dry more easily but tend to be more hygroscopic and stickier, which can potentially complicate handling. Therefore, it is necessary to select the appropriate maltodextrin DE level and balance the need for physical stability and ease of processing.

Some polysaccharides (chitosan, alginate, carboxymethylcellulose, etc.) are included in formulations for their mucoadhesive properties and/or to modify the drug release profile, which may be advantageous in the development of specific dosage forms, such as sustained-release oral products or inhalable powders.

## 4 From laboratory to industry

Laboratory-scale spray dryers provide a fundamental basis for early-stage formulation development, particularly when producing small quantities of powder for feasibility testing. These systems offer the advantage of processing minimal volumes of feeding material (as little as 2–5 mL) with relatively high yields, making them ideal for research and development (Poozesh and Bilgili, 2019; Diana et al., 2025). These compact spray dryers can even serve as valuable tools in developing new medicinal products through the pre-clinical and clinical phases, where low quantities are required. By operating continuously for extended periods, some advanced control spray dryers can produce up to several kilograms. In some cases, due to their adaptability, laboratory-scale spray dryers are used to manufacture low-volume commercial products.

Thanks to their glass chambers and cyclones, they allow for real-time observation of the drying and separation processes, providing critical insights into how the product behaves during spray drying. However, a significant challenge in scaling up from laboratory-scale to industrial spray drying systems lies in managing differences in drying kinetics, including evaporative capacity and residence time. The reduced dimensions of the drying chamber limit the residence time, necessitating smaller droplets that can dry completely before leaving the chamber (Huang et al., 2018). Consequently, many laboratory-scale spray dryers produce powders with particle sizes below 20 μm. There are exceptions, such as laboratory-scale dryers with extended tower designs (e.g., APT-2.0 from APTSol or the ProCepT unit), which can process larger droplets (60–100 μm), thereby enabling these systems to more closely mimic industrial-scale particle size and morphology. Such features are critical when transitioning from laboratory-scale to industrial-scale spray drying because they address the need for consistent drying kinetics and control over key parameters such as bulk density and relative saturation, which are known to influence particle morphology and solvent retention during the process (Thybo et al., 2008; Poozesh and Bilgili, 2019). These features allow the system to replicate the conditions of large-scale production more accurately, including enhanced control over drying kinetics, reduced risk of filter clogging, and improved handling of higher throughput while maintaining consistency in particle size and quality.

As development moves to the next stage, pilot- and commercial-scale spray dryers can process a wide range of batch sizes, from less than 1 kg to hundreds of kg. These units share many design similarities with laboratory-scale dryers, including configurations for different feed materials and the ability to handle a variety of organic solvents. The primary challenge in scaling up spray drying from lab to industrial scale is managing evaporation capacity and throughput, as larger systems must maintain consistent product quality while processing larger volumes. While laboratory-scale systems excel in producing small batches with precision, larger units must be carefully optimized to maintain product consistency at higher capacities. The evaporative capacity is influenced by key factors such as the drying gas flow rate, solvent type, temperature profiles (inlet and outlet temperatures), and heat loss in the system; differences in these parameters can lead to variations in particle size, morphology, and overall product quality, requiring careful calibration to achieve the desired outcomes during scale-up (Arpagaus, 2023).

To achieve successful scale-up, it is essential to leverage drying kinetics descriptors – such as bulk density, relative saturation, and drying rate – that remain valid across scales. By understanding these descriptors, it is possible to predict particle behavior and adjust process parameters to maintain product quality across varying production scales. As the process is scaled up, understanding how these parameters affect the drying kinetics and particle properties becomes crucial to maintaining product quality and consistency across different production scales.

In preparation for future scale-up, it is crucial to address common issues that may arise during production, such as material buildup on the nozzle and deposits on the drying chamber walls, which can necessitate frequent production stops and result in process inefficiencies. Additionally, the product's stability can pose challenges, potentially requiring frequent interruptions if not adequately addressed during the early stages of the formulation process. Proper control of process parameters, such as temperature and drying gas flow rate, is essential to prevent these issues and ensure consistent drying kinetics, thereby reducing the risk of production downtime (Gil et al., 2010; Poozesh and Bilgili, 2019). For these reasons, the initial R&D trials are critical, as they lay the groundwork for overcoming these challenges and ensuring a smoother scale-up in later production phases. Proper formulation and process optimization during this phase are crucial to achieving consistent and efficient large-scale operations.

## 5 Trends and modern applications

### 5.1 Evolution of spray drying for biopharmaceuticals and vaccines

Spray drying has progressively evolved from a classical industrial dehydration technique into a highly sophisticated process for stabilizing complex biopharmaceutical molecules. Its intrinsic capability for rapid solvent removal offers remarkable advantages in preserving the integrity, biological activity, and long-term stability of sensitive therapeutic entities such as proteins, peptides, nucleic acids, and biologics (Doerr et al., 2020; Sharma et al., 2021; Emami et al., 2023; Donthi et al., 2024). Unlike conventional drying techniques, spray drying operates on a rapid timescale, minimizing exposure of labile molecules to thermal degradation and facilitating the production of amorphous, homogeneous powders that can maintain functionality for extended periods. These properties have driven growing interest in spray drying across the pharmaceutical and biotechnological fields, especially where cold-chain dependency or liquid instability limits the practicality of conventional formulations (Bowen et al., 2013).

The first significant demonstration of spray drying's pharmaceutical potential was the development of Exubera®, an inhalable form of insulin pioneered by Pfizer (Stevenson and Bennett, 2014; Weers et al., 2019). Exubera® represented a paradigm shift by offering a non-invasive route of insulin administration, bypassing subcutaneous injections and improving patient compliance (Khan et al., 2022). Although it was withdrawn from the market in 2007 due to factors including the complexity of the inhalation device, patient training requirements, and limited commercial uptake, its introduction marked a critical milestone (Heinemann, 2008). The Exubera® case provided invaluable technological insights into particle engineering, aerodynamic optimization, and stability management that shaped the development of subsequent inhalable biopharmaceuticals.

Following this, the pharmaceutical industry expanded spray drying into more refined applications, with several successful inhalable biologics reaching commercialization. Afrezza®, approved by the Food and Drug Administration in 2014, exemplifies this evolution. Using Technosphere® technology, Afrezza® delivers ultra-rapid-acting insulin as a dry powder, improving pharmacokinetics and offering greater patient acceptability than injections (Richardson and Boss, 2007). Similarly, TOBI Podhaler®, a spray-dried tobramycin formulation, demonstrates how spray drying can convert aqueous antibiotic formulations into stable, respirable powders for treating pulmonary infections in cystic fibrosis (Geller et al., 2011). These products collectively highlight spray drying's ability to overcome traditional stability and delivery barriers, thereby transforming biologic administration via inhalation.

While inhalable formulations have reached maturity in commercialization, a more recent frontier involves injectable biologics and thermostable vaccines. Spray drying offers a unique opportunity to produce dry powders that are less sensitive to temperature fluctuations and can eliminate the stringent requirements of cold-chain distribution (Huang et al., 2022). This feature is desirable for resource-limited environments where infrastructure constraints impede the distribution of temperature-sensitive drugs. Nonetheless, the transition of spray drying from inhalable to injectable formulations introduces new technical and regulatory complexities. Injectable formulations must meet rigorous sterility and reconstitution standards, and the physicochemical stresses of atomization and drying can alter protein conformation, leading to aggregation or loss of bioactivity. Therefore, formulation strategies increasingly focus on excipient design – specifically sugars, amino acids, and surfactants – to protect against denaturation and interfacial stress during atomization.

In the vaccine sector, spray drying is emerging as a transformative approach to enhance thermostability and logistics flexibility. The COVID-19 pandemic underscored the fragility of mRNA-based vaccine platforms that rely on ultra-cold storage, as seen in the Pfizer-BioNTech, Moderna, and CureVac vaccines (Kanojia et al., 2017; Pinto et al., 2021; Arpagaus, 2023). Spray drying has since been intensively studied as a method for converting liquid formulations into solid powders, thereby mitigating dependence on the cold chain. Over the past two decades, the number of studies reporting enhanced dry vaccine stability has increased significantly. By 2021, approximately 26% of WHO-prequalified vaccines were available as dry powders, primarily produced by lyophilization. Although no spray-dried vaccine has yet achieved commercial approval, the technique offers several distinct advantages: improved heat tolerance, reduced refrigeration requirements, simplified transportation, and potential for alternative delivery routes, such as intranasal or inhalation-based immunization.

Current investigations focus on spray-dried vaccines targeting tuberculosis, influenza, and rotavirus, aiming to produce heat-stable formulations suitable for ambient storage. An auspicious direction concerns the spray drying of mRNA vaccines into thermostable powders intended for pulmonary or nasal delivery (Sarode et al., 2024). These developments could revolutionize vaccine distribution, particularly in regions with limited access to ultra-cold logistics. However, the shift toward spray-dried vaccines introduces unique challenges compared to traditional liquid or lyophilized counterparts. The intense thermal, shear, and interfacial stresses inherent to spray drying can compromise the structural integrity of lipid nanoparticles and the nucleic acids they encapsulate. These stresses may trigger RNA fragmentation, lipid oxidation, or phase separation, thus diminishing immunogenicity. Moreover, the high process throughput required for vaccine production poses challenges for ensuring uniform particle morphology and dose reproducibility.

Additional constraints involve process scalability, formulation costs, and post-processing requirements. High-precision equipment and stringent environmental controls are necessary to prevent contamination, particularly during the processing of live attenuated or recombinant vaccines. Furthermore, spray-dried vaccine powders often require reconstitution before administration, necessitating diluents and potentially complicating field use in resource-limited environments (Arpagaus, 2023). Therefore, the practical success of spray-dried vaccines depends on addressing these specific process and formulation challenges, particularly by optimizing excipient matrices that stabilize labile biomolecules and minimize shear-induced degradation.

In summary, spray drying has evolved from an industrial drying operation into a versatile platform for producing biopharmaceutical and vaccine powders. The method now occupies a critical position in the development of next-generation biologics, where formulation stability, patient convenience, and cold-chain independence converge. Yet, these advances come with new technological demands: maintaining molecular integrity under severe process stresses, ensuring sterility and uniformity in large-scale operations, and achieving regulatory acceptance for complex dry-powder formulations. Addressing these challenges through interdisciplinary optimization, linking process engineering, material science, and molecular stabilization, will define the future trajectory of spray drying in the biopharmaceutical sector.

### 5.2 Spray drying in nanomedicine: Engineering challenges and critical advances

Nanocarriers such as lipid nanoparticles (LNPs), nanostructured lipid carriers, cubosomes, and polymeric micelles have become essential tools in modern drug delivery, enabling the controlled release of bioactive molecules, enhanced solubility of poorly water-soluble drugs, and improved permeation across biological barriers (Almurshedi et al., 2021; Drago et al., 2021; Deruyver et al., 2023; Sipos et al., 2025). The convergence of nanotechnology with pharmaceutical engineering has positioned spray drying as a transformative technique for converting liquid nanosuspensions into physically robust, dry-powder formulations that preserve nanoscale characteristics. This transition addresses critical bottlenecks in the storage, transport, and administration of nanomedicines, particularly the instability of aqueous dispersions and their susceptibility to aggregation or hydrolysis during storage.

However, integrating nanocarrier systems into spray drying introduces distinct physicochemical and engineering challenges. Unlike conventional molecular drugs, nanocarriers exhibit structural complexity, interfacial sensitivity, and thermolability, which make them prone to exposure to heat, shear, and dehydration. The goal of spray drying in nanomedicine is therefore not only to produce powders with optimal flow and aerodynamic properties but also to ensure that the encapsulated nanoparticles retain their original physicochemical attributes, such as size, surface charge, and bioactivity, after reconstitution.

However, for specific routes of administration, spray-dried powders must meet specific requirements. For pulmonary delivery applications, aerosolized powders must exhibit aerodynamic diameters between 1 and 5 μm to achieve deposition in the deep lung regions. However, nanoparticles themselves are typically below 200 nm and would be exhaled if delivered as isolated entities. This necessitates the “nano-into-micro” engineering strategy, in which nanoparticles are embedded within microparticle matrices that facilitate aerodynamic deposition while preserving nanoscale functionality after rehydration (Quarta et al., 2021; Quarta et al., 2023). Following inhalation, these microspheres dissolve in lung fluids, releasing the constituent nanoparticles in their original, non-aggregated form.

The successful realization of this dual-scale design presents a complex interplay between formulation, droplet drying kinetics, and excipient selection. Spray drying must balance several competing requirements: (i) maintaining the aerodynamic size and morphology needed for deep lung delivery, (ii) preserving nanoparticle integrity against thermal and mechanical stresses, and (iii) ensuring complete redispersion into monodisperse nanosuspensions. The engineering precision required to control these attributes simultaneously distinguishes spray drying in nanomedicine from its use in conventional formulations.

Among the most critical technical hurdles is managing process-induced stresses that threaten nanoparticle integrity. During atomization, the liquid feed is subjected to high shear rates and rapid solvent evaporation, generating significant interfacial stresses and concentration gradients. For thermosensitive systems such as lipid-based carriers or nucleic acid-loaded nanoparticles, these stresses can induce structural collapse, lipid phase transitions, or degradation of encapsulated actives. For example, Zimmermann et al. (2022) investigated RNA-loaded lipid nanoparticles subjected to spray drying and demonstrated that process optimization was essential to retain functional bioactivity. Through careful adjustment of inlet/outlet temperatures and excipient composition, they achieved stable powders with minimal RNA degradation (<15%), preserved nanoparticle size (∼150 nm), and effective gene silencing (>95% knockdown).

These findings underscore the importance of defining a narrow operational window that accommodates both the physicochemical constraints of the nanocarrier and the thermodynamic demands of drying. To establish this window, formulation screening must precede process design, incorporating predictive thermal analysis and stability assays to determine safe drying conditions. Excipients such as sugars (e.g., trehalose, lactose) and polyols act as stabilizing matrices, forming glassy networks that encapsulate nanoparticles and minimize shear-induced deformation. Such “protective shell” approaches mitigate structural damage while enhancing the powder's redispersibility.

A second major challenge lies in preventing nanoparticle aggregation and simultaneously engineering the aerodynamic properties of the resulting microparticles. Aggregation typically arises from the rapid water loss and increased particle concentration at the end of droplet drying. Once nanoparticles come into close contact in a shrinking droplet, van der Waals and capillary forces can cause irreversible clustering. Thus, formulation design must ensure that excipients form an appropriate spatial barrier during solvent removal.

Excipients such as mannitol and leucine are particularly effective matrix formers that aid in dispersibility and particle engineering (Gordon et al., 2024). However, their ratios critically affect the post-redispersion size and uniformity of nanoparticles (Quarta et al., 2021; Quarta et al., 2023). Mannitol contributes to crystalline shell formation and moisture control, whereas L-leucine, a hydrophobic amino acid, rapidly migrates to the droplet surface, reducing cohesion and improving powder flowability (Berruyer et al., 2023). Morphological design, such as generating hollow, wrinkled, or corrugated structures, further improves aerosolization by reducing bulk density and enhancing the respirable fraction. The precise control of droplet solidification kinetics thus governs both particle aerodynamics and nanoscale preservation, illustrating the multi-scale engineering complexity unique to nano-into-micro spray drying.

Beyond the physical properties of the powders, an additional frontier in nanomedicine spray drying concerns biological targeting and barrier navigation. Upon deposition, nanoparticles must traverse biological barriers, particularly mucus and epithelial layers, without losing functionality. The design must therefore consider two opposing strategies: muco-inert versus muco-adhesive formulations.

For diseases such as cystic fibrosis, where thickened mucus severely impedes diffusion, muco-inert nanoparticles are essential. Chai et al. (2020) demonstrated that spray-dried muco-inert particles can maintain diffusion coefficients in human airway mucus and cystic fibrosis sputum comparable to those of their liquid precursors, indicating successful preservation of surface properties. Conversely, muco-adhesive systems exploit electrostatic or hydrogen-bonding interactions to enhance retention time and permeation. Chitosan-coated cubosomal nanoparticles, for example, exhibit strong interactions with negatively charged mucins due to their high positive zeta potential, resulting in a thousand-fold increase in apparent permeability compared with uncoated systems (Deruyver et al., 2023). Similarly, nano-spray-dried polymeric micelles modified with hyaluronic acid exhibit both mucoadhesive and hydrating effects, transiently loosening epithelial tight junctions and facilitating deeper tissue penetration (Sipos et al., 2025).

These dual strategies illustrate how spray drying is not only a drying step but a surface-engineering tool capable of preserving or inducing specific interfacial properties essential for biological performance. However, this also introduces new formulation constraints: maintaining surface-active functionalities during high-temperature drying and preventing their degradation or rearrangement at the air-liquid interface.

A further critical consideration in nanomedicine spray drying is long-term stability and control of cytotoxicity. Amorphous spray-dried powders, while often more soluble, are thermodynamically metastable and prone to moisture-induced crystallization or collapse. Residual humidity plays a decisive role in determining storage stability and redispersion performance. Maintaining residual water levels below 5% is generally necessary to preserve nanoparticle size and bioactivity.

In parallel, formulation safety must be balanced with therapeutic efficacy. Positively charged excipients used to promote cellular uptake or mucosal adhesion can increase cytotoxicity. Cationic lipids and polymers such as chitosan may disrupt cell membranes at higher concentrations. Thus, formulation design must carefully optimize charge density and excipient ratios to achieve sufficient biological interaction without compromising cell viability.

The inclusion of antioxidants and moisture scavengers can further stabilize powders by minimizing oxidative degradation, especially in lipid-based nanocarriers.

Despite its inherent complexity, spray drying has become an indispensable platform in nanomedicine development. Its unique ability to convert unstable nanosuspensions into dry, inhalable, or reconstitutable powders with tuneable aerodynamic and interfacial properties makes it a cornerstone for the next generation of drug delivery systems. Significantly, the new challenges it introduces, multi-scale process integration, nano-structural preservation under stress, surface chemistry retention, and cytotoxicity mitigation, are driving the evolution of both process engineering and formulation science.

Ultimately, spray drying in nanomedicine represents a powerful yet demanding intersection of materials science, thermodynamics, and biological engineering. The requirement to maintain nanoscale precision within a microscale delivery platform has transformed spray drying from a conventional process into an advanced, multi-dimensional technology capable of shaping the future of targeted and personalized drug delivery.

### 5.3 The future in aseptic spray drying

As spray drying expands its role in biopharmaceutical manufacturing, aseptic spray drying is emerging as a key technological frontier that bridges process efficiency with the stringent sterility demands of parenteral and inhalable biologics (Siew, 2016). Traditional pharmaceutical spray drying has primarily been conducted under non-sterile or semi-sterile conditions, with sterilization typically performed post-process through filtration, irradiation, or aseptic filling. However, the increasing use of highly sensitive biological products, such as monoclonal antibodies, peptides, nucleic acids, and vaccines, makes many of these downstream sterilization steps impractical or destructive. Consequently, aseptic spray drying aims to integrate sterility assurance directly within the drying process, thereby producing sterile powders suitable for immediate aseptic filling or direct administration without additional sterilization (Arpagaus, 2023).

Most spray dryers, operating under validated cGMP conditions in an ISO 7 or ISO 8 cleanroom, can produce powders with low bioburden but cannot meet sterile requirements.

Maintaining sterility in a continuously operating spray dryer presents multiple technical challenges. The most critical are:

1. Sterilization of the drying gas: the drying air or nitrogen must be filtered through high-efficiency sterile filters (often 0.22 μm pore size) to remove microorganisms. Gas heating elements must not compromise sterility or introduce particulates.
2. Sterile atomization: the atomizer, whether a two-fluid nozzle or rotary disk, must be sterilized before use and kept isolated from environmental contamination. Atomization systems are often the weakest link in aseptic design because they involve high-speed moving parts and complex geometries that are difficult to sterilize.
3. Closed powder collection: powder must be collected in sterile containers under laminar flow or directly filled into vials within an aseptic isolator. Each transfer point introduces potential contamination risk.

**Fig. 10:**
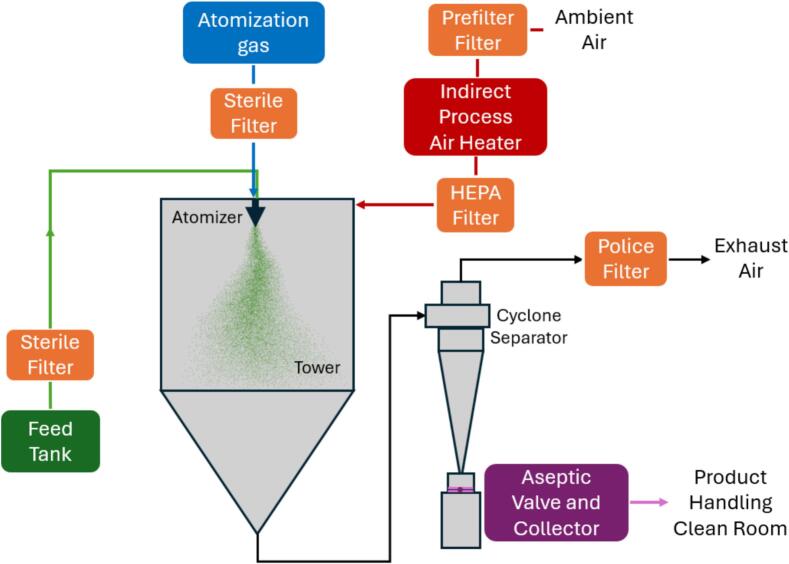
Graphical scheme of an aseptic spray drying system.

In this scenario, although the core components resemble those of standard equipment described in previous chapters, an aseptic spray dryer must be specifically designed and manufactured for sterile operation. The design must accommodate cleanroom requirements, incorporating sterile filters and containment systems. To ensure that both air and equipment remain contaminant-free, spray drying typically relies on sterile air filtration and closed-loop systems- to maintain aseptic conditions (Fig. 10). This became feasible with advances in High-Efficiency Particulate Air (HEPA) filters. The first description of an aseptic spray dryer appeared in 1975 (Masters and Vestergaard, 1975), while the world's first aseptic spray drying plant (the Cambridge Biostability plant) for the cGMP production of sterile vaccines was opened in 2009, in Leicester (UK).

To increase the lifetime of HEPA filters, prefilters are usually installed before the inlet gas utilities, and bag filters are placed for the exhaust process gas before the outlet HEPA filter. The drying gas inlet HEPA filter is positioned after the heater, ensuring that any contaminants are retained before entering the drying chamber. The feeding solution is pumped through an inline sterile filter; for suspensions, a sterile preparation is required to maintain aseptic conditions upstream in the process. While HEPA filters are essential for filtering the air entering the system, the performance and integrity of both HEPA and sterile-grade filters for the liquids must be validated. The validation process typically involves multiple tests, including the bubble point test, the DOP test (also known as the DEHS test), and leak tests of system seals. The bubble point test is commonly used to validate sterile filters, such as those with a 0.22 μm pore size, to ensure that no microorganism can penetrate the filter. This non-destructive test is performed by saturating the filter with a low-surface-tension liquid (e.g., water or isopropyl alcohol) and applying compressed air to the upstream side. The pressure is gradually increased until air passes through the largest pore, forming the first bubble, which is detected by a pressure sensor. The resulting bubble point pressure is compared against the manufacturer's specifications to confirm filter integrity; if the measured bubble point meets the expected value, the filter is considered intact (Arpagaus, 2023). For HEPA filters, which are designed to remove at least 99.97% of particles as small as 0.3 μm, a different validation method is employed: the DOP test, also known as the DEHS test (using Diethylhexyl Sebacate as the aerosol), is used to verify their efficiency. This test is typically performed in situ, where an aerosol of DEHS particles is injected into the air stream upstream of the HEPA filter. Temporary sampling points are used to measure particle concentration upstream and downstream of the filter using a photometer. The device measures the particle concentration to determine whether the filter effectively traps the aerosol. Ensuring the integrity of the system's seals and gaskets is equally crucial, as even a small leak can compromise sterility. Leak tests are performed to detect air or gas leaks at critical points where seals are used, such as doors, flanges, and connection points. Usually, a pressure-decay test is performed, in which the system is pressurized, and any drop in pressure over time indicates a potential leak.

The whole equipment must then adhere to strict standards for surface finishes, materials, and design to ensure cleanliness, sterilizability, and corrosion resistance. According to ASME BPE standards, surfaces in contact with the product must have a maximum roughness average (*Ra*) of 0.38 μm, with options for mechanical polishing or electropolishing; the latter offers enhanced smoothness and minimizes contamination risks. Moreover, the equipment must feature rounded edges and welds free of gaps to prevent product residue build-up and ensure efficient cleaning. Materials like 316L stainless steel are preferred for their high corrosion resistance and compatibility with passivation and electropolishing treatments, which further protect against corrosion and improve cleanability. The number of components in contact with the product should be minimized to reduce the risk of leaks, particularly critical in piping systems.

The design must ensure full inspectability of the internal surfaces by operators to facilitate maintenance and cleaning. Additionally, the process should be maintained at a slightly positive pressure to prevent external contaminants from entering the system (0.069 bar) (Ohtake et al., 2020). Although biologics are mostly aqueous solutions, suspensions, or emulsions that can be processed in open spray dryer configurations, one of the most widely used approaches is to operate with nitrogen in a closed loop to eliminate the potential risk of contamination, even though this increases cost. This may also be helpful if the vaccine needs protection from oxidation. An appreciated solution, especially at pilot-scale and larger sizes, is the “through the wall” configuration of the equipment, which can maintain all the equipment's technical areas and utilities separated from the process and clean product contact areas (Arpagaus, 2023).

Regarding the sterilization process, while on a small scale, disassembly and sterilization can be done at the component level, on a large scale, the equipment must also be compatible with in-situ sterilization processes, making it suitable for repeated cleaning and sterilization cycles, which are essential for maintaining aseptic conditions. The methods currently used include: i) steam in place (SIP), ii) dry heat, iii) vaporized hydrogen peroxide (VHP), and iv) vaporized ethylene oxide (VEtO). Currently, SIP sterilization is the standard approach, which involves exposing the inner surface of the equipment to 121–135 °C under 2–3 bar for at least 20 min. However, it has some downsides; the higher pressure required means that the equipment's welds and pressure-rated structure must withstand these conditions, which in turn requires thicker steel and additional structural reinforcements, making the equipment noticeably heavier. Special system pressure sensors, condensate traps, and additional thermal insulation are often required. No thread parts must be exposed to the SIP to eliminate crevices that could harbor microorganisms and evade sanitization. The steam and high pressure generated during the SIP procedure must also be conveyed through the feed line, atomization gas line, process gas line, nozzle, and filters, as well as the spray drying chamber and its associated piping. Moreover, this technique must be accompanied by surface washing with sterile water; indeed, the SIP process itself cannot depyrogenate or remove pyrogens, such as endotoxins (Arpagaus, 2023).

As the spray dryer is a type of drying equipment that operates under heating conditions, the traditional approach involves applying dry heat to sterilize parts that come into contact with the product. In this case, a higher temperature of 170 °C needs to be maintained across the internal surfaces for 2–4 h to ensure that the final Sterility Assurance Level (SAL) is 10^−6^ as requested by the Eudralex Volume 4, Annex 1 (EU GMP Guidelines for the Manufacture of Sterile Medicinal Products) (EudraLex, 2025). Complete depyrogenation, even if possible, would also pose a challenge in this case due to the low heating capacity of dry gas and the difficulty of maintaining the inner surface at the right temperature. The presence of cold spots (e.g., at the junction) necessitates an oversized principal heater capable of effectively heating even the far surfaces, albeit at a significant energy consumption. For this reason, extra insulation or additional heating blankets are installed along the pipes and components. This procedure may be suitable for small equipment, where compact design enhances heating efficiency and facilitates temperature monitoring (Ohtake et al., 2020).

In the context of pharmaceutical and biotechnological sterilization, VHP and VEtO are two key methods employed for biodecontamination. VHP is increasingly favored due to its short cycle times, low-temperature operation, and non-toxic by-products (water and oxygen), making it ideal for sterilizing pharmaceutical isolators, filling lines, and medical devices (Meleties et al., 2023). VHP effectively eliminates a broad spectrum of microorganisms, achieving over a 6-log reduction in bioburden. However, it is primarily a surface sterilant with limited penetration, which can pose challenges for reaching narrow tubes or cavities. For this reason, vacuum cycles are used to remove air and enhance the effectiveness of VHP sterilization, allowing the vapor to penetrate more thoroughly into cavities and ensuring a more uniform distribution, thereby increasing the likelihood of effective sterilization. In contrast, EtO excels in sterilizing materials with deep or complex structures, such as lumens and tubing, due to its superior penetration. However, its use is declining due to significant health and safety concerns, including carcinogenicity, flammability, and the need for extensive aeration to remove toxic byproducts like ethylene glycol and ethylene chlorohydrin. Despite its effectiveness in sterilizing temperature-sensitive equipment, regulatory limitations and its environmental impact make it a less viable option for widespread use (Gustin, 2021). The increasing adoption of VHP in aseptic environments aligns with the industry's move toward safer and more sustainable sterilization methods. Nonetheless, VHP persistence of residual hydrogen peroxide poses a significant risk, particularly to the quality of protein therapeutics, which are susceptible to oxidation. For this reason, to ensure no residual VHP remains, the procedure validation should be conducted with particular attention to the aeration phase (Meleties et al., 2023).

**Fig. 11:**
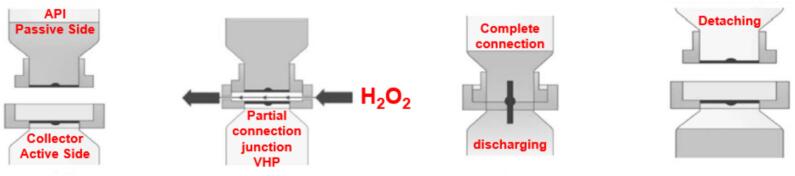
Aseptic valve functioning procedure.

Finally, in aseptic spray dryers, the sterile handling of powders requires careful management to prevent contamination. The dried product is usually collected in a stainless-steel charge bottle, which is stored in Grade D, ISO 8 conditions. These bottles undergo a SIP process before being moved to the spray dryer clean room in Grade C, ISO 7. Product transfer in aseptic conditions is possible using systems such as split butterfly valves, which ensure that the transfer occurs under Grade A, ISO 5 protection conditions. The operation sequence begins with the sterilization of the active half using a SIP or VHP procedure. Then, the two halves are locked and sealed together, exposing the internal surfaces to decontamination gas. The butterfly valve is then opened to allow powder transfer, with both the active and passive interfaces sealed to prevent infiltration. After the transfer, the units are safely decoupled, maintaining the system's sterility (Fig. 11). These special butterfly valves are preferred over ball valves in these processes because they allow for a more compact, efficient design that is easier to clean and sterilize. Unlike ball valves, butterfly valves do not trap as much powder residue, reducing the risk of contamination. Some authors also report the feasibility of using pinch valves for powder discharge, but their use in aseptic conditions poses challenges. The flexible rubber material, which is effective for contaminant-free discharge, is more challenging to sterilize than the metal surfaces of butterfly valves, as rubber may harbor contaminants and degrade during sterilization. Ensuring sterility with pinch valves requires careful SIP processes, but the risk of contamination remains high. Ultimately, butterfly valves are generally preferred for aseptic powder handling due to their rigid design, which facilitates easier sterilization and provides better protection in continuous production environments. However, butterfly valves are in-motion mechanical parts, and may also pose a risk of contamination due to friction and wear, potentially releasing particles into the process (Arpagaus, 2023).

The physical properties of the spray-dried powders, such as particle size, density, and morphology, must be carefully controlled to ensure that the product is not only sterile but also retains its biological activity. Lastly, because spray drying exposes biopharmaceuticals to stresses such as heat and atomization, careful optimization of process parameters is essential to prevent the degradation of sensitive proteins and peptides while maintaining sterility (Donthi et al., 2024). The shear stress induced by the atomization can provoke the loss of about 15% of the bioactivity, because, during atomization, proteins are exposed to high shear rates, reaching up to 105 s^−1^, particularly as they pass through the nozzle orifice. However, protein degradation is primarily driven by the synergistic effects of shear stress and interfacial stress at the air-liquid interface. This interface creates a large surface-to-volume ratio, which promotes protein unfolding and aggregation, leading to instability. To mitigate these effects, optimizing atomization parameters, such as lowering the spray pressure, adjusting the feeding flow rates, and refining the nozzle design, can help reduce degradation. Additionally, the inclusion of stabilizers in the formulation is recommended to protect protein integrity during atomization, thereby ensuring product stability (Barceló-Chong et al., 2024b; Moino et al., 2024).

Overall, aseptic spray drying is viewed as an emerging technology that has the potential to catalyze research. For this reason, several inventions and patents have been addressed to improve the feasibility of aseptic spray drying systems and processes for the production of dried pharmaceutical and active biological ingredients (Arpagaus, 2023).

In the coming years, the success of aseptic spray drying will depend on overcoming key hurdles: ensuring complete sterilization of closed systems, maintaining the biological integrity of thermolabile molecules, enabling safe and automated powder handling, and achieving regulatory harmonization. If these challenges are met, aseptic spray drying could redefine the manufacturing paradigm for biologics, offering a continuous, sterile, and energy-efficient alternative to lyophilization.

As global healthcare moves toward more temperature-sensitive and high-value biologics, the demand for aseptic, scalable, and stable dry-powder formulations will continue to rise. Aseptic spray drying thus stands at the frontier of pharmaceutical engineering, a technology not only of adaptation but of transformation, poised to bridge the gap between laboratory innovation and industrial reality in next-generation biopharmaceutical production.

## 6 Conclusions

Spray drying is a well-established and versatile technology that has long served as a cornerstone of powder production across diverse industrial sectors. Its ability to transform liquids into stable, free-flowing powders with controlled size, morphology, and moisture content has made it indispensable in the manufacture of high-quality products. While traditional challenges – such as the processing of heat-sensitive compounds or sugar-rich formulations – have historically limited its scope, technological innovations in equipment design, process optimization, and formulation strategies have significantly expanded its applicability.

In recent decades, spray drying has undergone a profound transformation, moving beyond its traditional roles in the food and chemical industries to become a platform technology in biotechnology and pharmaceuticals. It now plays a central role in the development of inhalable therapies, amorphous solid dispersions, and controlled-release systems, while emerging applications in biopharmaceutical stabilization, vaccine formulation, and nanomedicine highlight its capacity to address some of the most pressing challenges in modern drug delivery. The parallel rise of aseptic spray drying further underscores its potential in producing sterile powders for parenteral administration and next-generation vaccines.

Looking ahead, research efforts focused on energy efficiency, scalability, and sustainability will continue to strengthen spray drying's relevance in industrial production. At the same time, innovations in particle engineering and aseptic processing are expected to unlock new therapeutic frontiers. With its unique combination of versatility, precision, and adaptability, spray drying is poised not only to retain but to expand its pivotal role as an enabling technology for the future of biotechnology and pharmaceutical manufacturing.

## CRediT authorship contribution statement

**Andrea Milanesi:** Writing – original draft, Visualization, Methodology, Conceptualization. **Giada Diana:** Writing – review & editing, Validation. **Alessandro Candiani:** Writing – review & editing, Visualization. **Alessandro Sodano:** Writing – review & editing. **Paolo Rassè:** Writing – review & editing. **Andrea Foglio Bonda:** Writing – review & editing, Conceptualization. **Elia Bari:** Writing – review & editing, Visualization. **Maria Luisa Torre:** Writing – review & editing, Visualization. **Lorena Segale:** Writing – review & editing, Writing – original draft, Supervision, Project administration, Funding acquisition, Conceptualization. **Lorella Giovannelli:** Writing – review & editing, Visualization, Supervision.

## Funding

The present work is part of the project “PROMISE–Produzione di microparticelle Spray Dried con Secretoma da cellule Mesenchimali per la Rigenerazione Polmonare e il wound healing” (Id. 0200106) funded by Programma di Cooperazione INTERREG Italia-Svizzera 2021–2027.

## Declaration of competing interest

The authors declare the following financial interests/personal relationships which may be considered as potential competing interests: Lorena Segale reports financial support was provided by Regione Lombardia, Programma INTERREG IT-CH. Lorena Segale reports a relationship with APTSol that includes: board membership. Lorella Giovannelli reports a relationship with APTSol that includes: board membership. Andrea Milanesi reports a relationship with APTSol that includes: board membership and employment. Paolo Rasse reports a relationship with APTSol that includes: board membership and employment. Alessandro Sodano reports a relationship with APTSol that includes: employment. Andrea Foglio Bonda reports a relationship with APTSol that includes: board membership and employment. If there are other authors, they declare that they have no known competing financial interests or personal relationships that could have appeared to influence the work reported in this paper.

## Data Availability

No data was used for the research described in the article.
